# An explanatory evo-devo model for the developmental hourglass

**DOI:** 10.12688/f1000research.4583.2

**Published:** 2014-12-18

**Authors:** Saamer Akhshabi, Shrutii Sarda, Constantine Dovrolis, Soojin Yi

**Affiliations:** 1 School of Computer Science, Georgia Institute of Technology, Atlanta, GA, 30332, USA; 2 Center for Bioinformatics and Computational Biology, University of Maryland, College Park, MD, 20740, USA; 3 School of Biology, Georgia Institute of Technology, Atlanta, GA, 30332, USA

## Abstract

The "developmental hourglass'' describes a pattern of increasing morphological divergence towards earlier and later embryonic development, separated by a period of significant conservation across distant species (the "phylotypic stage''). Recent studies have found evidence in support of the hourglass effect at the genomic level. For instance, the phylotypic stage expresses the oldest and most conserved transcriptomes. However, the regulatory mechanism that causes the hourglass pattern remains an open question. Here, we use an evolutionary model of regulatory gene interactions during development to identify the conditions under which the hourglass effect can emerge in a general setting. The model focuses on the hierarchical gene regulatory network that controls the developmental process, and on the evolution of a population under random perturbations in the structure of that network. The model predicts, under fairly general assumptions, the emergence of an hourglass pattern in the structure of a temporal representation of the underlying gene regulatory network. The evolutionary age of the corresponding genes also follows an hourglass pattern, with the oldest genes concentrated at the hourglass waist. The key behind the hourglass effect is that developmental regulators should have an increasingly specific function as development progresses. Analysis of developmental gene expression profiles from *Drosophila melanogaster* and *Arabidopsis thaliana *provide consistent results with our theoretical predictions.

## Introduction

The evolutionary mechanism of conservation during embryogenesis, and its connection to the gene regulatory networks that control development, are fundamental questions in systems biology ^1– 3^. Several models have been presented in the context of morphological, molecular, and genetic developmental patterns. The most widely discussed model is the “developmental hourglass”, which places the strongest conservation across species in the “phylotypic stage”. The first observations supporting the hourglass model go back to von Baer when he noticed that there exists a mid-developmental stage in which embryos of different animals look similar ^4^. On the other hand, the “developmental funnel” model of conservation predicts increasing diversification as development progresses ^5, 6^.

Recently, the hourglass model has come under new light. Multiple studies have observed the hourglass pattern across diverse biological processes, including transcriptome divergence ^7– 12^, transcriptome age ^7, 13, 14^, molecular interaction ^15^, and evolutionary selective constraints ^10, 15, 16^. Despite these observations the genomic basis and even the existence of the developmental hourglass effect have been the subject of an intense debate ^1, 6, 14, 17– 22^. More importantly, the underlying mechanism that can shape the developmental process in the hourglass or funnel forms is still unknown.

We aim to understand the conditions under which the hourglass effect can emerge in a general setting, based on an abstract model for the evolution of embryonic development. The model focuses on a hierarchical network that represents the temporal “execution” of the underlying Gene Regulatory Network (GRN) during development. Each layer of the network corresponds to a developmental stage. The nodes at each layer represent regulatory genes (i.e., genes encoding transcription factors or signaling molecules) that undergo significant activity change at that corresponding stage. The edges from genes at one layer to genes at the next layer represent regulatory interactions that cause those activity changes. We refer to this hierarchical network as Developmental Gene Execution Network (DGEN) to distinguish it from the corresponding GRN. A DGEN is subject to evolutionary perturbations (e.g., gene deletions, rewiring, duplication) that may be lethal, or that may impede development, for the corresponding organism.

The model predicts that the evolutionary process shapes the DGENs of a population in the form of an hourglass, under fairly general assumptions. Specifically, the number of genes at each developmental stage follows an hourglass pattern, with the smallest number at the “waist” of the hourglass. The main condition for the appearance of the hourglass pattern is that the DGEN should gradually get sparser as development progresses, with general-purpose regulatory genes at the earlier developmental stages and highly specialized regulatory genes at the later stages. This assumption is motivated from the well established patterns of increasing modularity as the embryo develops ^23^. Under the previous condition, the model predicts that gene regulatory changes or rewiring in mid-development are more likely to cause cascades of removing non-essential genes from the DGEN, compared to early or late developmental stages. Another model prediction is that the evolutionary age of DGEN genes also follows an hourglass pattern, with the oldest genes concentrated at the waist.

We have examined the aforementioned predictions using transcriptome data from the development of *Drosophila melanogaster* and *Arabidopsis thaliana*. This data is insufficient to reconstruct the complete DGEN of these species but it allows to estimate the number of genes at each developmental stage, given an activity variation threshold. Under a wide range of this threshold, the inferred DGEN shape follows an hourglass pattern, the waist of that hourglass roughly coincides with the previously reported phylotypic stage for these species, and the age of the corresponding genes follows the predicted hourglass pattern.

### Developmental gene execution networks

As a first-order approximation, a regulatory gene can be modeled in one of several discrete *functional states* ^24^. In the simplest case, a regulatory gene can act as a binary switch (“on” and “off”) but in general a gene may have more than two functional states. The transition of a regulatory gene X from one functional state to another is often (but not always) caused by one or more upstream regulators of X that go through a functional state transition before X. We use the term *transitioning gene* to refer to a regulatory gene that goes through a functional state transition at a given developmental time anywhere at the developing embryo.

**Figure 1.**
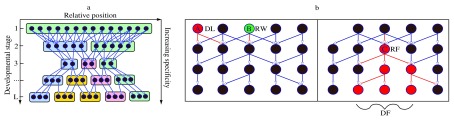
An abstract DGEN. ( **A**) The circles denote state-transitioning genes, edges represent directed regulatory interactions, and colored boxes refer to spatial domains that form during development. If regulatory genes become increasingly function-specific as development progresses, the network gradually becomes sparser in that direction. ( **B**) Evolutionary perturbations on a DGEN’s structure: Gene A is deleted (DL), while gene B is rewired (RW) losing an outgoing edge. This RW event causes the regulatory failure (RF) of gene C, which then causes a cascade of five more RF events. This cascade causes developmental failure (DF). Note that the removal of some upstream regulators does not always cause an RF event (e.g., genes regulated by A).

A DGEN is a directed and acyclic network; see Figure 1a for an abstract example. The vertical direction refers to developmental time, from the zygote at the top to the developed organism at the bottom. In the horizontal direction we can represent different spatial domains, even though this is not necessary and it is not done in our model. For instance, the zygote at the top of the DGEN would be a single domain, while the organism at the bottom of the DGEN would have the largest number of spatial domains.

Development is often approximated (conceptually and experimentally) as a succession of discrete developmental stages. The duration of a developmental stage can be thought of as the typical time that is required for a gene’s functional state transition, and it does not need to be the same for all stages. Each layer of a DGEN refers to a developmental stage, and it includes only the transitioning genes during that stage anywhere in the embryo. The same gene can appear in more than one stage if it goes through several functional state transitions during development. Additionally, a DGEN edge from a gene *X* at stage *l* to a gene *Y* at stage *l* + 1 implies that the functional transition of *X caused* the functional transition of *Y* at the next stage. If gene *Y* has more than one incoming edge, its functional state transition was caused by the coupled effect of more than one transitioning genes at the earlier stage. Any upstream regulators of *Y* that remained at the same functional state at stage *l* are not included in that stage of the DGEN.

The sequence of developmental stages is denoted by *l* = 1 . . . *L*. The set of transitioning genes at stage *l* is *G*( *l*). A gene *g* at stage *l<L* regulates a set of *downstream* genes at stage *l*+1 denoted by *D*( *g*) (outgoing edges from g). Similarly, a gene *g* at stage *l>*1 is regulated by a set of *upstream* genes *U*( *g*) at stage *l*-1 (incoming edges to g). The functional transitions at the first stage are assumed to be caused by regulatory maternal genes that initiate the developmental process.

### Model description

The model captures certain aspects of both the developmental process, in the form of a given DGEN for each embryo, and of the evolutionary process, as random perturbations in the structure of individual DGENs in a population. The model does not need to capture the actual functional state transitions or the regulatory input function of each gene. It does capture however the dynamic and stochastic effect of structural network perturbations (gene deletion, rewiring and duplication) on the success of the developmental process, as explained in the following.

Similar to the Wright-Fisher model ^25^, we consider a population of *N* individuals, each represented by a DGEN. In each generation, individuals reproduce asexually, inheriting the DGEN of their parent. Various evolutionary events can cause structural changes in the DGEN of an individual that may result in “developmental failure”. Such individuals (and their DGENs) are replaced with developmentally successful individuals so that the population size remains constant.

The model is meant to be as general as possible and so the regulatory interactions between genes of successive stages are determined probabilistically, as follows. Each stage *l* is assigned a *regulatory specificity*, or simply *specificity s*( *l*) with 0 ≤ *s*( *l*) ≤ 1. A gene *g* at stage *l* acts as upstream regulator for a gene *g′* at stage *l* + 1 with probability *s*′( *l*) = 1 − *s*( *l*). So, the specificity of a developmental stage determines how likely it is for regulatory genes of that stage to cause a state transition of the genes at the next stage; a higher specificity decreases that likelihood.

Our major assumption is that the regulatory specificity increases substantially as development progresses. In other words, the DGEN becomes gradually sparser along the developmental time axis, with *s*(1) << *s*( *L*). This assumption is plausible for the following reasons. First, as development progresses the embryo grows in size forming distinct spatial domains. So, extracellular gene regulation becomes more difficult, especially across different domains. Additionally, as development progresses the transitioning genes become more organ- or tissue-specific, implying that their downstream interactions become sparser. Unfortunately, a direct and empirical investigation of the increasing specificity assumption requires knowledge of the complete DGEN for a given species; this is currently not feasible for even the most well-studied model organisms. However, this assumption is plausible if we consider the well established patterns of increasing modularity as the embryo develops at the morphological, signaling, and genomic levels ^23^.

The DGEN structural changes we consider are gene deletions, gene duplications, and gene rewiring:

**Deletions (DL):** This event removes a gene from the DGEN, including its incoming and outgoing edges. There are many genetic mechanisms that may cause such events. A DL event deletes each gene of an individual and at each generation with probability *P _DL_*.

**Duplication (DP):** This event creates an identical copy of a gene *g* with the same downstream and upstream regulators and at the same developmental stage as *g*. The two genes may have different fates if one of them is subject later to deletion or rewiring. Otherwise, the two genes are considered identical. A DP event duplicates each gene of an individual and at each generation with probability *P _DP_*.

**Rewiring (RW):** This event changes the upstream and/or downstream regulators of a gene. Changes in the upstream versus downstream regulators may have different biological basis. The former occur, for instance, as a result of mutations in the transcription factor binding sites in a gene’s promoter or mutations in distal regulatory elements such as enhancers, while the latter may be mostly caused by coding sequence mutations. The details of the rewiring process do not affect the results qualitatively as long as the average density of edges in each stage remains consistent with the specificity of that stage. The specific rewiring mechanisms we use are presented next.

Suppose that a RW event affects gene *g* at stage *l*. The upstream regulators of *g* are recomputed based on the specificity of the previous stage, i.e., by choosing each distinct gene of stage *l* −1 with probability *s′*( *l* −1). For the downstream regulators of *g*, we randomly remove *N _–_* existing outgoing edges of *g*, and then add *N*_+_ outgoing edges to randomly chosen genes of stage *l* + 1 that *g* is not already connected to. If *B*( *N*, *p*) denotes a Binomial random variable with *N* trials and success probability *p*, the random variables *N*_−_ and *N*_+_ are independent and they both follow the *B*(| *D*( *g*)|, *s*′( *l*)) distribution (| *X*| denotes the cardinality of set *X*). Thus, the number of downstream edges of gene *g* after a Rewiring event becomes:

              | *D _new_*( *g*)| = | *D*( *g*)| − *N _–_*+ *N*_+_,

which is at least 0 and at most 2 × | *D*( *g*)|. This captures that the downstream regulators of *g* are derived by incremental changes in *D*( *g*), instead of giving *g* a completely new network configuration (thereby, new regulatory function). The higher the regulatory specificity of a stage, the less likely these incremental changes are. An RW event rewires each gene of an individual and at each generation with probability *P _RW_*.

A gene deletion or rewiring event at stage *l* can remove an upstream regulator from genes at stage *l* + 1. A loss of incoming edges may trigger the *regulatory failure* of a gene, as described next.

**Figure 2.**
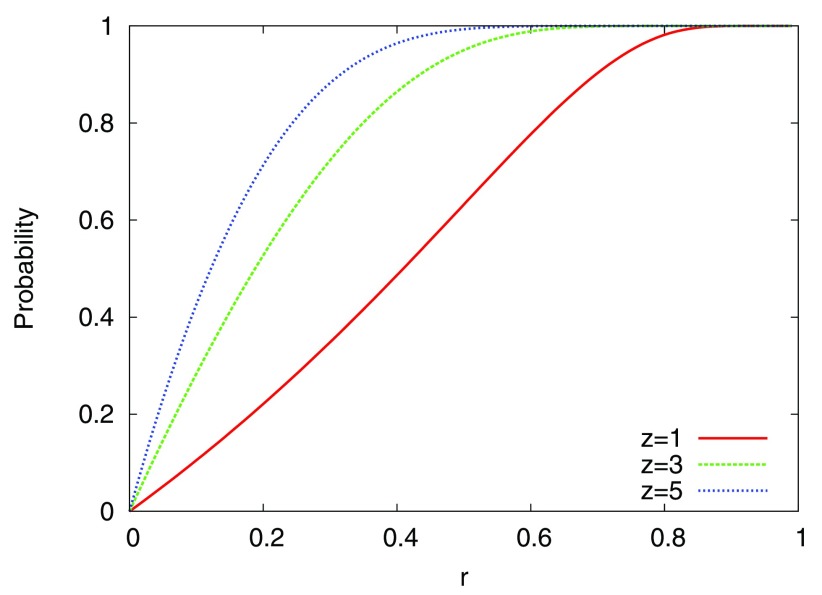
Probability of Regulatory Failure (RF) for three values of the parameter *z*. *r* is the fraction of upstream regulating edges that are lost due to a DL or RW event.

**Regulatory failures (RF):** A gene *g* may not be able to change functional state if some of its upstream regulators *U*( *g*) are lost due to DL or RW events (see Figure 1b). Even though regulatory networks are often robust to structural perturbations, even a partial gene loss in *U*( *g*) may disable *g* causing a *regulatory failure*. It is plausible that the probability of a regulatory failure increases with the fraction of lost upstream regulators. So, if *U*′( *g*) is the new set of upstream regulators and | *U*( *g*)| *>* | *U*′( *g*)| *>* 0, gene *g* is removed with probability:

$$P_{RF}(r)=1-e^{\frac{-zr}{1-r}},\;\;0<r=1-\frac{\left|U'(g)\right|}{\left|U(g)\right|}<1$$

while if | *U*′( *g*)| = 0 we set *P _RF_* (1) = 1. *z* is the *RF parameter* and it depends on the robustness of regulatory interactions to gene loss (see Figure 2).

When a DL or RW event causes one or more RF events, the latter can trigger additional RF events in subsequent developmental stages, leading to *cascades of regulatory failures*. Such RF cascades may cause *developmental failure*, meaning that the developed embryo is unable to survive or reproduce.

**Developmental failure (DF):** The last stage of a DGEN represents the fully developed embryo. If that stage includes Γ transitioning genes at a successfully developed embryo, the simplest assumption is that an individual with less than Γ genes at stage- *L* has failed to develop properly. Such DGENs are removed from the population and they are replaced with randomly chosen but successfully developed DGENs. We have also experimented with two variations of the DF event: first, the individual is removed if its last stage has less than Γ − *γ* genes, where *γ* is small relative to Γ, and second, the probability of a DF event increases as the number of genes at stage- *L* decreases below Γ. The qualitative results, as described next, do not change with these two model variations.

## Methods

**Hourglass score H.** Suppose that *w*( *l*) denotes the number of transitioning genes in stage *l*, also referred to as the width of stage *l*. Let *b* be the stage with the minimum number of such genes. We construct the sequences *X* = { *w*( *l*), *l* = 1, . . . *b*} and *Y* = { *w*( *l*), *l* = *b*, . . . *L*}. *τ _X_* and *τ _Y_* denote the normalized univariate Mann-Kendall statistic for monotonic trend in each sequence, respectively ( *τ* is -1 for decreasing, +1 for increasing and almost 0 for random sequences) ^26^. The H score is defined as

$$H=\frac{\tau_{Y}–\tau_{X}}{2}.$$

**Figure 3.**
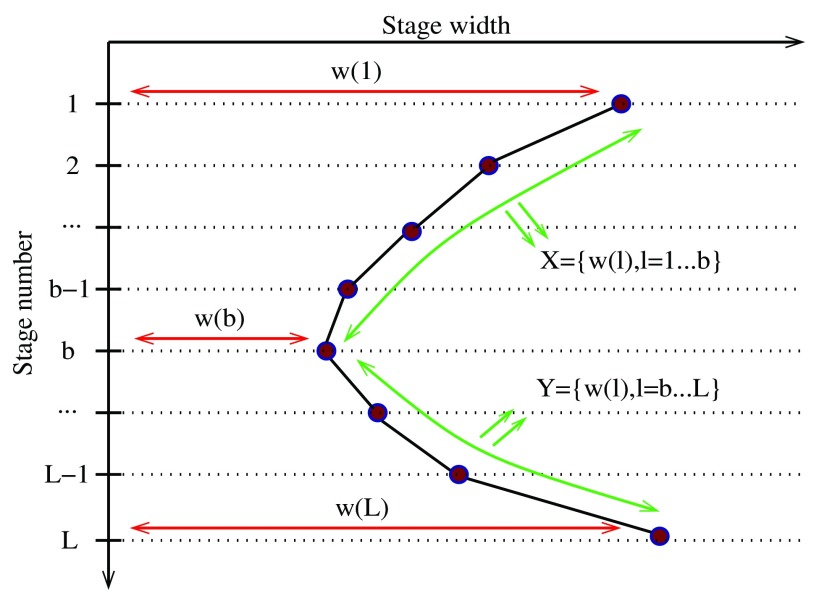
Illustration of the *H* score calculation. Let *w*( *l*) be the width of stage *l*. Let *w _b_* be the minimum width across all stages, and suppose that this minimum occurs at stage *l* = *b*; this is the *waist* of the network (ties are broken so that the waist is closer to ⎣ *L/*2⎦). Consider the sequence *X* = { *w*( *l*)}, *l* = 1, . . . *b*} and the sequence *Y* = { *w*( *l*)}, *l* = *b*, . . . *L*}. We denote the normalized univariate Mann-Kendall statistic for monotonic trend on the sequences *X* and *Y* as *τ _X_* and *τ _Y_*, respectively. The Mann-Kendall statistic varies between -1 (decreasing) and 1 (increasing), and it is approximately zero for a random sequence. We define *H* = ( *τ _Y_* − *τ _X_*) */*2; *H* is referred to as the hourglass score. *H* = 1 if the DGEN is structured as an hourglass, with a decreasing sequence of *b* stages followed by an increasing sequence of *L* − *b* stages. In the computational modeling results, we do not consider the width of the first stage because it can never decrease in Models-1,2,3. We also define a variation of the hourglass score in which we do not take into account adjacent stages in calculating the two Mann-Kendall statistics. That statistic is denoted by $\tilde{H}$ and is referred to as the robust hourglass score.

See Figure 3 for an illustration, and for the definition of a more robust version of H.

**Statistical analyses.** All biological data processing and analyses were performed using custom scripts written in Java (JDK v1.6) [Dataset 14].

**Drosophila data and treatment.** Developmental gene expression profiles for *D. melanogaster* are obtained from two sources. First, we obtained microarray data from Kalinka *et al.* ^9^ for 3,610 genes. The expression level of each gene is calculated as the median of probes mapping to that gene. Each stage represents a 2-hr interval during the first 20 hours of embryogenesis (10 stages). The second source is RNA-Seq data from Graveley *et al.* ^27^. Raw data are processed to RPKM values. Each stage represents a 2-hr interval during the first 24 hours (12 stages). Genes with zero RPKM value in all stages are discarded, resulting in 14,110 genes.

**Arabidopsis data and treatment.** Genome-wide expression profiles of a complete developmental series from the zygote to the mature embryo in *A. thaliana* were obtained from Xiang *et al.* ^28^. This comprised of microarray expression levels for 25,207 genes across seven developmental stages. Signal background correction and normalization of raw expression values was carried out by Xiang *et al.* ^28^.

**Transitioning gene identification.** Suppose that the reported expression value of gene *i* at stage *l* is *e _i_*_, *l*_. We analyze both these “absolute” expression values as well as the normalized expression values, given by

$${\mathrm{e\prime}}_{i,l}=\frac{e_{i,l}}{\sum_{j}e_{j,l}}$$

The identification of transitioning genes follows the same method for both absolute and normalized expression levels. In the case of absolute expressions we define *δ _i,l_* = *e _i,l_*− *e _i,l–_*_1_ for each gene *i* and at each stage *l* = 2 . . . *L*, while in the case of normalized expressions, we similarly define *δ _i,l_* = *e*′ _*i,l*_ − *e*′ _*i,l–*1_. Gene *i* is considered “transitioning” at the stage-pair ( *l* − 1, *l*) if

                 | *δ _i,l_* | *> c*,

where *c* is a given transition threshold. This condition is more robust to noise than a ratio-based rule ( *e*′ _*i,l*_/ *e*′ _*i,l–*1_) for the identification of transitioning genes. Note that a gene may be transitioning at more than one stage-pair, but it may also not be transitioning at any stage-pair.

**Figure 4.**
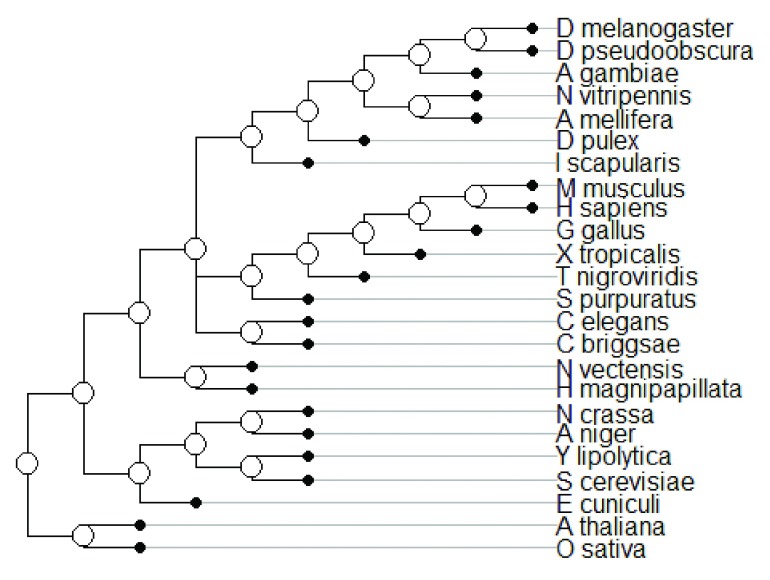
The phylotypic tree that we use to calculate the age index of *Drosophila*’s genes. Each gene is assigned to one of the following six ages: Level-1: Common ancestor to Fungi, Plants and Eumetazoa. Level-2: Common ancestor to Fungi and Eumetazoa. Level-3: Common ancestor to all Eumetazoa. Level-4: Common ancestor to all Bilateria. Level-5: Common ancestor to all Arthropoda. Level-6: Common ancestor to all Dipteria.

**Transcriptome age index (TAI).** We collected the groups of orthologs for each gene in Drosophila using two databases, OrthoDB ^29^ and OrthoMCL ^30^. The Eumetazoa data were taken from OrthoDB, while Fungi and Plants species were retrieved from OrthoMCL, and the two datasets were merged. Using these orthologs we then assigned an age index to each gene based on its absence and presence in a phylogenetic tree of 24 well-diverged species (see Figure 4) ^11, 13, 31^ [Dataset 7].

The transcriptome age index (TAI) values for Arabidopsis genes were obtained from ^11^.

**Age index for each stage-pair.** Suppose that we identify transitioning genes based on the normalized expression levels, and that *n*( *l*) genes are assigned to stage-pair ( *l* − 1, *l*). Denote by *p _i_* the phylogenetic rank (TAI value) of gene *i*. The age index assigned to that stage-pair is given by

$$\text{TAI}(l)=\frac{\sum {}_{i=1}^{n(l)}p_{i}e{'}_{i,l}}{\sum {}_{i=1}^{n(l)}e{'}_{i,l}}.$$

The same method is used when transitioning genes are identified based on absolute expression levels.

## Results

### Simulation

We simulate the presented model to examine the properties of the surviving DGENs as evolutionary time progresses. The initial population consists of *N* identical DGENs with Γ genes at each stage. The edges between genes are constructed probabilistically based on the specificity of each stage, as described previously. Simulating the complete model would not show the significance of individual mechanisms such as the increasing specificity assumption. For this reason we construct a sequence of four models with increasing complexity, presenting results separately for each of them:

**Model-1: Constant specificity.** Each stage has the same specificity, *s*( *l*) = 0.5 for *l* = 1 . . . *L* − 1. Further, this model does not include gene deletion and duplication. Gene rewiring can cause RF and DF events even if there are no DL or DP events. We have also experimented with other values of the specificity probability, and the results are qualitative the same (namely, when the specificity is constant there is no hourglass effect).

**Model-2: Increasing specificity.** The difference from Model-1 is that the specificity is gradually increasing across developmental stages. Unless noted otherwise, the specificity is linear, *s*( *l*) = *l/L* for *l* = 1 . . . ( *L* − 1); a nonlinear specificity function is also considered, which we describe later. We do not claim that these particular specificity functions are realistic or that there are experimental results that suggest them. They are just the simplest and most parsimonious models that lead to the emergence of the hourglass pattern.

**Model-3: With gene duplications.** Model-3 adds DP events in Model-2. The duplication probability *P _DP_* is set so that the average size of a DGEN, across the entire population, stays within a given range (70%–80% of *L* × Γ genes).

**Model-4: With gene deletions.** Model-4 adds DL events in Model-3 (complete model). The deletion probability *P _DL_* is set so that the average size of a DGEN, across the entire population, stays within the same range as in Model-3.

In Model-1 and Model-2, genes can be only removed (due to RW events, potentially followed by RF cascades) and so the average DGEN size decreases as evolutionary time progresses, which is unrealistic. Model-3 and Model-4 are more realistic because they can maintain a roughly constant DGEN size in the long-term. However, as will be shown next, all aspects of the developmental hourglass effect can already be seen with Model-2 (but not with Model-1). This highlights that the increasing specificity assumption is sufficient to generate the hourglass effect. Further, the inclusion of additional biological mechanisms in the model, namely gene duplication and gene deletion, even though they make the model more realistic, they are not necessary for the emergence of the hourglass effect.

**Figure 5.**
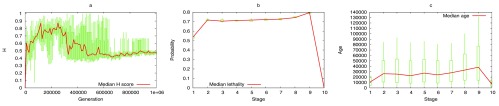
Computational results with Model-1. Parameters: 10 runs with different initial populations, *N* = 10 individuals, *L* = 10 stages, specificity function *s*( *l*) = 0.5 for all stages, Γ = 100 genes at each stage initially, RF parameter *z* = 4, 1,000,000 generations, probability of RW event *P _RW_* = 10 ^−4^. The red line is the median and the green boxes are the 10th, 25th, 75th, and 90th percentiles, across all individuals and all runs. ( **a**) The hourglass score *H* across evolutionary time. ( **b**) Lethality probability at each stage. ( **c**) Age of existing genes at the last generation.

**Figure 6.**
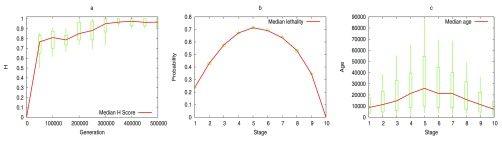
Computational results with Model-2. Parameters: 10 runs with different initial populations, *N* = 1000 individuals, *L* = 10 stages, specificity function *s*( *l*) = *l/L* ( *l* = 1 . . . *L* − 1), Γ = 100 genes at each stage initially, RF parameter *z* = 4, 500,000 generations, probability of RW event *P _RW_* = 10 ^−4^. The red line is the median and the green boxes are the 10th, 25th, 75th, and 90th percentiles, across all individuals and all runs. ( **A**) The hourglass score *H* across evolutionary time. ( **B**) Lethality probability at each stage. ( **C**) Age of existing genes at the last generation.

**Figure 7.**
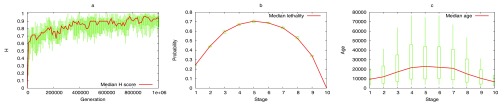
Computational results with Model-3. Parameters: 10 runs with different initial populations, *N* = 10 individuals, *L* = 10 stages, specificity function *s*( *l*) = *l/L* ( *l* = 1 . . . *L* − 1), Γ = 100 genes at each stage initially, RF parameter *z* = 4, 1,000,000 generations, probability of RW event *P _RW_* = 10 ^−4^. The probability of gene duplication *P _DP_* is adjusted dynamically so that the average DGEN size stays between 700 and 800 genes. The red line is the median and the green boxes are the 10th, 25th, 75th, and 90th percentiles, across all individuals and all runs. The hourglass score *H* across evolutionary time. ( **b**) Lethality probability at each stage. ( **c**) Age of existing genes at the last generation.

**Figure 8.**
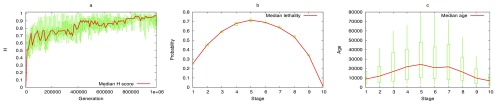
Computational results with Model-4. Parameters: 10 runs with different initial populations, *N* = 10 individuals, *L* = 10 stages, specificity function *s*( *l*) = *l/L* ( *l* = 1 . . . *L* − 1), Γ = 100 genes at each stage initially, RF parameter *z* = 4, 1,000,000 generations, probability of RW event *P _RW_* = 10 ^−4^. The probability of gene duplication *P _DP_* is adjusted dynamically so that the average DGEN size stays between 700 and 800 genes. The probability of gene deletion (DL) is *P _DL_* = 10 ^−6^. The red line is the median and the green boxes are the 10th, 25th, 75th, and 90th percentiles, across all individuals and all runs. ( **a**) The hourglass score *H* across evolutionary time. ( **b**) Lethality probability at each stage. ( **c**) Age of existing genes at the last generation.

**Hourglass shape.** A first observation is that as evolutionary time progresses, DGENs acquire an “hourglass-like” shape in Models-2,3,4. This means that the width of each stage first decreases until a certain stage (referred to as the waist of the hourglass) and then gradually increases. The hourglass may not be symmetric with respect to the waist. To quantify this observation, we define an “hourglass score” *H* (see Methods and Figure 3) that is equal to 1 if the sequence of *L* stage widths consists of two segments: a decreasing sub-sequence of *k* ≥ 2 stages followed by an increasing sub-sequence of *L* − *k* ≥ 2 stages. Figure 6a shows the hourglass score for the population of DGENs in Model-2. Similar graphs for the three other models are shown in Figure 5a, Figure 7a, and Figure 8a. The *H* score quickly increases in the three models that have increasing specificity, and it fluctuates close to 1 afterwards.

What is the reason behind the hourglass shape of DGENs? When a gene *g* is rewired at stage *l*, it may trigger RF events in stage *l* + 1 depending on the number of its lost outgoing edges. In the first few stages, where specificity is low, it is unlikely that a gene would lose a large fraction of its (typically many) incoming edges. In the last few stages, where specificity is high, edges are unlikely to get rewired in the first place. In the mid-stages however, where the specificity is close to 50%, there is higher variability in the number of outgoing edges lost or gained due to RW events. The loss of several outgoing edges due to an RW event at stage *l* can trigger several RF events and gene removals in the subsequent stage. Thus, the probability of RF events in mid-stages is higher than in early/late stages, making the removal of genes more likely in the former.

The constant specificity of Model-1 does not result in an hourglass pattern [Dataset 1] (see Figure 5a) for the following reason. RW events at stage *l* can cause RF events at the next stage with the same probability, independent of *l*. However, after the occurrence of an RF event, the size of the potential cascade increases as *l* decreases simply because there are more subsequent stages to affect. This gives DGENs a “funnel-like” shape with a gradually increasing number of genes after stage-1; *H* fluctuates around 0.5, as expected for an increasing sequence.

**Stage lethality.** Another aspect of the developmental hourglass is in terms of the significance of each stage for the survival of the embryo. We define *lethality of stage l* as the probability that a RW or DL event at stage *l* starts a RF cascade that eventually leads to a DF event. We estimate this probability at generation *i* as the fraction of RW and DL events, during the past *i* generations, that occurred at stage *l* and led to a DF.

In Model-1, there is no clear trend for the stage lethality probability (see Figure 5b); with the exception of the last stage (in which RW events cannot result in gene loss), the lethality probability is roughly the same at all stages. In the three models with increasing specificity, however, we observe a clear pattern: the lethality gradually increases until the waist of the hourglass, and then it decreases [Dataset 2, Dataset 3 and Dataset 4] (see Figure 6b, Figure 7b, and Figure 8b). The reason, as explained earlier, is that the probability of RF events in mid-stages is higher that in early/late stages. Additionally, after the formation of the hourglass shape the mid-stages have relatively few genes and so an RF event in those genes is more likely to initiate a lethal RF cascade.

These computational results for the lethality probability across development are consistent with the empirical observations of Galis and Metz ^15^ about the increased mortality of rodents due to perturbations in the phylotypic stage (see also Discussion section).

**Age of genes.** A third aspect of the developmental hourglass effect is related to the evolutionary age of genes. The age of a gene *g* at generation *i* is defined as

*A*( *g*) = *i* − *t*_0_( *g*),

where *t*_0_( *g*) is the generation at which *g* was most recently rewired (and 0, if it was not rewired earlier). The rationale behind this definition is that a rewiring event may give that gene a new function, at least in terms of its upstream and downstream regulators ^32, 33^.

In the case of Model-2, Figure 6c shows the median age of the genes at each stage, considering the population of all genes across all individuals at a given generation. See Figure 5c, Figure 7c, and Figure 8c for the same results with the three other models. The evolutionary age at stage *l* follows the same pattern as the lethality probability: it gradually increases until we reach the waist of the hourglass, and then it gradually decreases. Genes at intermediate stages tend to be older because, as explained earlier, they are fewer and their rewiring is more likely to be lethal. When one of those genes *g* is rewired or deleted from a DGEN, the corresponding individual is often replaced (DF event) by another individual that has the same gene *g*. So, the genes at the waist of a DGEN tend to be more conserved than genes at earlier or later stages.

**Location of waist.** What controls the location of the hourglass waist in the developmental process?

**Figure 9.**
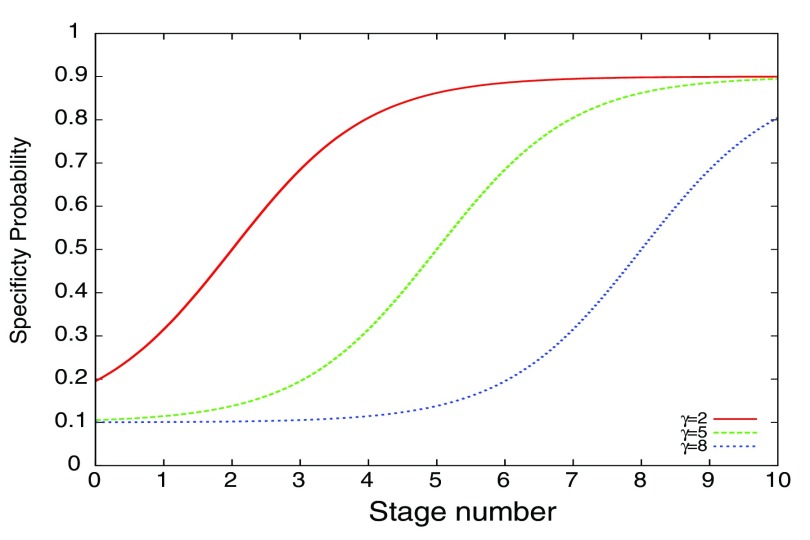
A nonlinear specificity function, *s*( *l*) = 0.9 − 0.8 */*(1 + *e ^γ−l^*), for three values of the parameter *γ*. This function allows us to control the stage *γ* at which the specificity is 50%.

**Figure 10.**
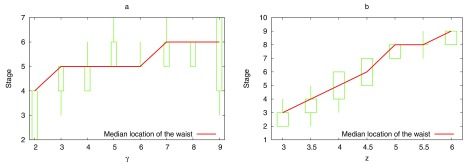
We examine the effect of the two model parameters that affect the location of the DGEN hourglass waist. The first is the specificity function. To examine its effect, we use a sigmoid-like mathematical function that controls the stage *γ* at which the specificity is 50% (see Figure 9). This is the stage with the maximum variance in the number of outgoing regulatory edges. RW events at this stage can cause the largest extent of rewiring and so, the highest likelihood of RFs in genes of the next stage. Graph ( **a**) shows that the location of the hourglass waist is “pushed” towards stage *γ*, even though it is not always exactly at that stage. The second way to affect the location of the hourglass waist is the parameter *z* that controls the shape of the RF probability. Increasing *z* makes RF events more likely, also increasing the likelihood of lethal RF cascades. Graph ( **b**) shows that as we increase *z* the hourglass waist moves towards later developmental stages. These results are obtained using Model-2. Parameters: 10 runs with different initial populations, *N* = 1000 individuals, *L* = 10 stages, specificity function *s*( *l*) = *l/L* ( *l* = 1 . . . *L* − 1), Γ = 100 genes at each stage initially, RF parameter *z* = 4, 500,000 generations, probability of RW event *P _RW_* = 10 ^−4^. The graphs show the median (red lines) and the 10th, 25th, 75th, and 90 ^th^ percentiles (green boxes) of the location of the waist.

The location of the waist is mostly affected by two parameters of the model: the shape of the specificity function and the RF parameter *z*. To examine the effect of the former we use the nonlinear function shown in Figure 9. *γ* is the stage at which the specificity is 50%, and so that stage has the maximum variance in the number of outgoing regulatory edges. RW events at this stage can cause the largest extent of rewiring and so, the highest likelihood of RFs in genes of the next stage. Figure 10a shows that the location of the hourglass waist is ”pushed” towards stage *γ*, even though it does not coincide always with that stage. Parameter *z* controls the shape of the RF probability: increasing *z* makes RF events more likely, also increasing the likelihood of lethal RF cascades. Figure 10b shows that as we increase *z* the hourglass waist moves towards later developmental stages [Dataset 5].

**Figure 11.**
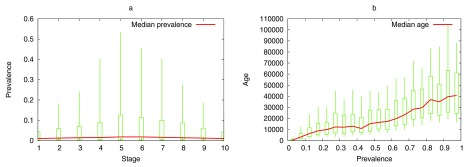
The prevalence of a gene *g* in a population of *N* individuals is the fraction of individuals in which gene *g* appears. These results are obtained using Model-2. Parameters: 10 runs with different initial populations, *N* = 1000 individuals, *L* = 10 stages, specificity function *s*( *l*) = *l/L* ( *l* = 1 . . . *L* −1), Γ = 100 genes at each stage initially, RF parameter *z* = 4, 500,000 generations, probability of RW event *P _RW_* = 10 ^−4^. The graphs show the median (red lines) and the 10th, 25th, 75th, and 90th percentiles (green boxes) for: ( **a**) prevalence of genes in each stage after 500,000 generations, and ( **b**) gene age as a function of gene prevalence. As expected, older genes tend to be more prevalent in the population.

**Gene prevalence.** We introduce a “gene prevalence” metric for gene *g* at time *t* as the fraction of individuals that include *g* at evolutionary time *t* [Dataset 6]. Figure 11a shows the gene prevalence metric across developmental stages after 500,000 generations, while Figure 11b shows the relation between gene prevalence and gene age. The genes at the waist of the developmental hourglass are not only the oldest but also the most prevalent across the population. The implication of this simulation result is that we can expect that those genes that are transitioning near the waist of the developmental hourglass will be the most conserved genes in a population.

### Data analysis

**Figure 12.**
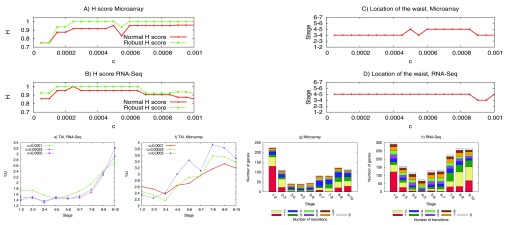
*Drosophila* results using normalized expression levels. Graphs ( **A**) and ( **B**) show the hourglass score (normal and robust) as a function of the transition threshold *c* for the two datasets. Graphs ( **C**) and ( **D**) show the location of the hourglass waist (stage-pair) as a function of the transition threshold *c* for the two datasets. Graphs ( **E**) and ( **F**) show the Transcriptome Age Index of transitioning genes for three different values of *c* (chosen so that the number of genes with known age index assigned to each stage is at least 25) for the two datasets. Graph ( **G**) shows the transitioning genes for the Microarray dataset with *c* = 0.0005. The transitioning genes constitute 11% of all genes in that dataset. 53% of those genes transition in a single stage-pair. Of the remaining, 64% transition only in consecutive stage-pairs. Note that if a gene transitions *n* times, it is counted in *n* stage-pairs. Similarly, graph ( **H**) shows the transitioning genes for the RNA-Seq dataset with *c* = 0.00025. The transitioning genes constitute 5% of all genes in that dataset. 45% of those genes transition in a single stage-pair. Of the remaining, 52% transition only in consecutive stage-pairs.

**Figure 13.**
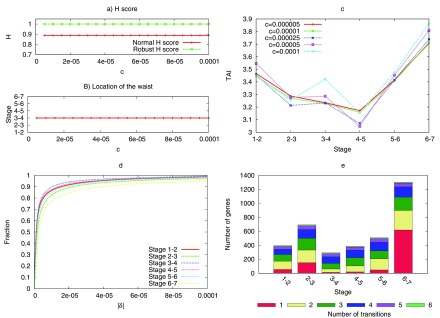
*Arabidopsis thaliana* results using normalized expression levels. Graph ( **a**) shows the hourglass score (normal and robust) as a function of the transition threshold *c*. Graph ( **b**) shows the location of the hourglass waist (stage-pair) as a function of the transition threshold *c*. Graph ( **c**) shows the Transcriptome Age Index of transitioning genes for five different values of *c* (chosen so that the number of genes with known age index assigned to each stage-pair is at least 290). Graph ( **d**) shows the CDFs of the expression level absolute variations | *δ*| across successive stage-pairs. Note that in this case the hourglass waist (in terms of number of transitioning genes) appears in stage-pair (3,4), while the oldest genes appear in the next stage-pair. Graph ( **e**) shows the transitioning genes with *c* = 0.0001. The transitioning genes constitute 7% of all genes in that dataset. 49% of those genes transition in a single stage-pair. Of the remaining, 36% transition only in consecutive stage-pairs.

We have examined the predictions of the previous model using transcriptome data for *Drosophila melanogaster* and *Arabidopsis thaliana* [Dataset 14]. We summarize results from both species here; the corresponding figures for which are Figure 12 and Figure 13 [Dataset 8 and Dataset 9]. For *Drosophila*, we analyze Microarray ^9^ and RNA-Seq ^27^ temporal expression profiles during the first 20 hours of development, taken at 10 stages of 2-hr intervals. We examine whether a) the number of transitioning genes follows an hourglass pattern, b) the waist of that hourglass coincides with the *Drosophila* phylotypic stage, and c) the evolutionary age of the transitioning genes follows a similar hourglass pattern. The two datasets are described in more detail in the Methods section. With such limited data, we cannot infer the regulatory edges between transitioning genes and we cannot reconstruct the underlying DGEN. However, we can identify the transitioning genes at each developmental stage given a “transition threshold” *c* (see Methods). Even though the correct value of this threshold is not known, the following results are robust in a wide interval of *c*, which includes most of the expression variation range across successive developmental stages (see Figure S3 for the CDFs of expression level variations across successive stages [Dataset 12]).

**Figure S1.**
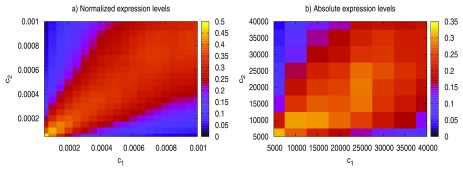
We evaluated the agreement between the two *Drosophila* datasets in terms of the transitioning genes assigned to each stage-pair, considering only those genes that appear in both datasets. Because the appropriate transition threshold may be different at each dataset, we use a different threshold for each dataset, say *c*_1_ and *c*_2_. For each pair ( *c*_1_, *c*_2_), we determine the transitioning genes at each stage-pair with the corresponding dataset., *L* − 1 pairs of gene sets), and then calculate the average Jaccard similarity across these *L* − 1 pairs. The Jaccard similarity maps show this average across all stage-pairs for various threshold pairs ( *c*_1_, *c*_2_). In graph ( **a**) with normalized expression levels, when the two thresholds are roughly equal, the average Jaccard similarity is as high as 50%; this means that about 2/3 of the genes assigned to a certain stage-pair using one dataset are also assigned to the same stage-pair using the other dataset. Graph ( **b**) shows a similar Jaccard similarity map for the case of absolute expression levels. [Dataset 10].

**Figure S3.**
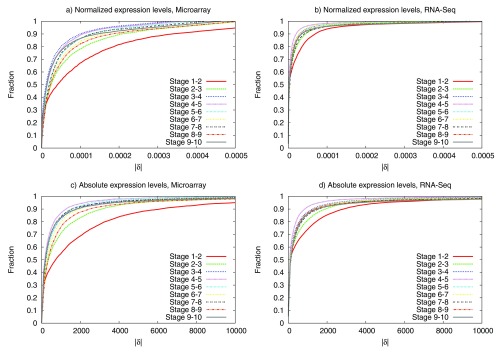
*Drosophila* data: CDFs of the expression level absolute variations | *δ*| across successive stage-pairs. ( **a**) normalized expressions, Microarray, ( **b**) normalized expressions, RNA-Seq, ( **c**) absolute expressions, Microarray, absolute expressions, RNA-Seq. [Dataset 12].

Figure 12a and Figure 12b show the hourglass resemblance score H (and its more robust variant) as function of *c*. Note that the H score is close to 1 for a wide range of *c*, confirming the presence of an hourglass-like structure in terms of the number of transitioning genes. Figure 12g and Figure 12h exhibit this pattern more clearly in the number of transitioning genes for a specific value of *c*. The two datasets also show reasonable agreement in terms of the assignment of transitioning genes to stage-pairs [Dataset 10] (see Figure S1).

Second, the location of the waist in this hourglass pattern, shown in Figure 12c and Figure 12d, occurs at the stage-pair (3,4) or (4,5), depending on *c*. This is roughly 8 hours after the formation of the zygote, and it includes the phylotypic stage for *Drosophila melanogaster* ^9^.

We have also estimated the evolutionary age of most of the transitioning genes at each developmental stage-pair using the Transcriptome Age Index (TAI) metric ^11^ (see Methods). TAI is lower for older genes. Figure 12e and Figure 12f show the average TAI for transitioning genes, weighted by the expression level of each gene, at each stage-pair and for each dataset using three values of *c*. The TAI index follows the pattern that the model predicts, with older genes (lower TAI values) close to the waist of the hourglass. This result appears consistent with the main observation of Domazet-Loso and Tautz ^13^, even though that study did not analyze transitioning genes.

The same approach was extended using transcriptome data ^28^ and TAI profiles ^11^ from *Arabidopsis*. The results obtained from the analysis ( Figure 13) were consistent with the predictions of the model, as well as the results obtained from Drosophila data.

## Discussion

Early studies of the developmental hourglass effect mostly analyzed morphological and phenotypic similarities across species ^2, 34^. Recently, the focus has shifted towards genomic and molecular comparative studies ^8, 9, 11, 13, 20^ that investigate conservation of gene expression variation, sequence conservation, selective constraint on coding sequences, and evolutionary gene “age”. These studies often report contradicting observations: some support strong conservation in earlier developmental stages ^5, 6, 20^, while others support that strongest conservation occurs at a mid-developmental stage ^7– 11, 13– 16^. Nevertheless, the fact that the hourglass effect is observed in highly divergent species across deep phylogenetic scales (including fish, flies and plants), suggests that this observed pattern of conservation may stem from fundamental organization principles.

What these principles are has remained elusive. Earlier stages may be conserved because any changes therein could have large cascading effects in later stages ^5, 35, 36^. Later stages may experience less constraint because as development progresses gene interactions become more modular, and so it is plausible that perturbations there have only local effects ^1^. We refer to them as the “temporal” constraint model and the “spatial” constraint model, respectively, following Tian *et al.* ^37^.

In this paper, we developed an evolutionary model of development that combines some aspects of the previous two models. Regulatory perturbations at a certain stage can cause cascades of regulatory failures at subsequent stages (temporal model), while the likelihood that a gene regulates genes at a subsequent stage decreases as development progresses (spatial model).

Our computational results lead to the following testable predictions: a) the number of transitioning genes during development follows an hourglass pattern, b) the evolutionary age of the transitioning genes also follows an hourglass pattern, with the oldest genes being at the waist of the hourglass, and c) the genes at the waist of that hourglass are the most essential, in the sense that their deletion maximizes the probability of developmental failure.

We have relied on developmental gene expression profiles of *Drosophila melanogaster* and *Arabidopsis thaliana* to examine the predictions of the model. The analysis of that data agrees with the first two theoretical model predictions. The third prediction of the model is in direct agreement with the empirical observations of Galis and Metz regarding the increased mortality caused by perturbations during the phylotypic stage ^15^. That study has shown, based on the teratological literature for rodent development, that disturbances in the phylotypic stage lead to much higher mortality than in other stages. Further, such disturbances lead to the co-occurrence of several distinct anomalies in the developing embryo and so the increased mortality cannot be due to a single particularly vulnerable process that takes place at that stage. Further, our simulations confirm that the details of these regulatory perturbations, such as the probabilities of gene duplication and deletion or the parameter *z* in the regulatory failure probability, do not affect the results of the model, at least at the qualitative level.

It is interesting to examine our results in the context of Raff’s hypothesis ^1^. Raff proposed an interesting explanation for the developmental hourglass effect based on the notions of “interconnectivity between body elements” and “developmental flexibility”. Specifically, his hypothesis states that the level of interaction (interconnectivity) between the elements of the developing embryo is maximized in mid-development. Early development is flexible because it governs robust and general global patterning processes, late development is also flexible because “signaling events within the primordia are little influenced by events in other primordia”, while mid-development (phylotypic stage) is least flexible because of the “high interconnectivity between elements that will later come to represent separate modules”. This hypothesis may be viewed initially as a contradiction with our increasing specificity assumption, which states that the density of regulatory interactions is maximized at the earliest stages of development. Note however that Raff’s hypothesis was not stated in terms of gene regulatory interactions – he was referring more generally to “developmental flexibility”. If we interpret the notion of “developmental flexibility” as the ability of an embryo to survive gene mutations and rewiring at different stages of the developmental process, then Raff’s hypothesis is actually consistent with our results regarding the “lethality probability” at each developmental stage (see Figure 6-B, Figure 7-B and Figure 8-B). The lethality probability is maximized at the phylotypic stage, and it is significantly lower at early and late developmental stages, following exactly the same pattern with Raff’s developmental flexibility.

The use of DGENs in this work was only as an abstract tool to study the effect of gene regulatory perturbations in the developmental process. In future work, it is important to infer the actual DGEN of model organisms. This will require information about gene regulatory interactions across time and space, but it should be possible for at least some developmentally well studied species ^24^. Such DGENs would help to identify the specific genes that form the hourglass waist and their function. Additionally, an inferred DGEN would allow to directly test the increasing specificity assumption.

Finally, we note that the hourglass effect (sometimes referred to as the ”bow-tie” effect) has also been observed in other complex biological and technological systems that exhibit hierarchical modularity and that are subject to evolutionary pressure or optimization tradeoffs ^38– 41^. Recently, Friedlander *et al.* have proposed a different mechanism for the emergence of hourglass-like patterns in evolving biological or technological networks, based on a linear system formulation ^42^. They showed that if a system can be represented as a hierarchical linear transformation of an input vector to an output vector, and the desired transformation matrix is rank-deficient, then an evolutionary process that selects that particular transformation can, under certain conditions, converge to an hourglass-like structure. It is not clear yet how to adapt this linear model in the context of inherently non-linear systems, such as gene regulatory networks.

Another example of a hierarchical system that is structured as an hourglass is the Internet “protocol stack” ^43^; this pattern was not designed but it emerged through the competition between protocols that serve roughly the same function at each communication layer, during the last 30–40 years. In earlier work, we proposed an abstract model (EvoArch) that captures the evolution of protocol architectures and that predicts the emergence of an hourglass structure. Interestingly, both EvoArch and the model of this paper share the same principle: the underlying hierarchical networks that control both systems should be increasingly sparser as complexity increases, i.e., the specificity of each complexity stage (or layer) should be increasing. In the future, we will further investigate this common organization principle between biological and technological systems.

## Data availability

The data referenced by this article are under copyright with the following copyright statement: Copyright: © 2014 Akhshabi S et al.

Data associated with the article are available under the terms of the Creative Commons Zero "No rights reserved" data waiver (CC0 1.0 Public domain dedication). 

“ZENODO: An evo-devo model for the developmental hourglass: Code and data”, DOI: 10.5281/zenodo.10579 ^44^, License: GNU GPLv3.

## Figures and Tables

**Figure S2.**
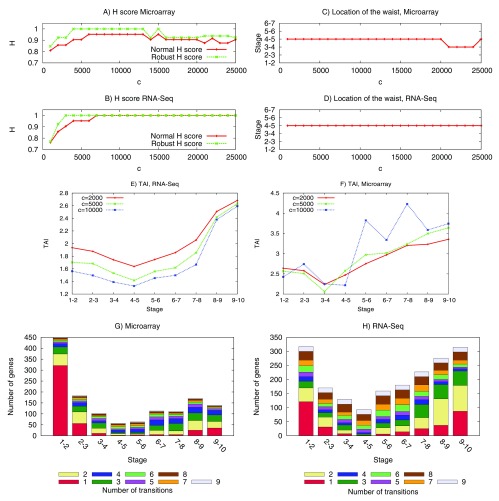
*Drosophila* results using absolute expression levels. Graphs ( **a**) and ( **b**) show the hourglass score (normal and robust) as a function of the transition threshold *c* for the two datasets. Graphs ( **c**) and ( **d**) show the location of the hourglass waist (stage-pair) as a function of the transition threshold *c* for the two datasets. Graphs ( **e**) and ( **f**) show the Transcriptome Age Index of transitioning genes for three different values of *c* (chosen so that the number of genes with known age index assigned to each stage is at least 10) for the two datasets. Graph shows the transitioning genes for the Microarray dataset with *c* = 5000. The transitioning genes constitute 21% of all genes in that dataset. 62% of those genes transition in a single stage-pair. Of the remaining, 58% transition only in consecutive stage-pairs. Similarly, graph ( **h**) shows the transitioning genes for the RNA-Seq dataset with *c* = 10000. The transitioning genes constitute 5% of all genes in that dataset. 45% of those genes transition in a single stage-pair. Of the remaining, 53% transition only in consecutive stage-pairs. [Dataset 11].

**Figure S4.**
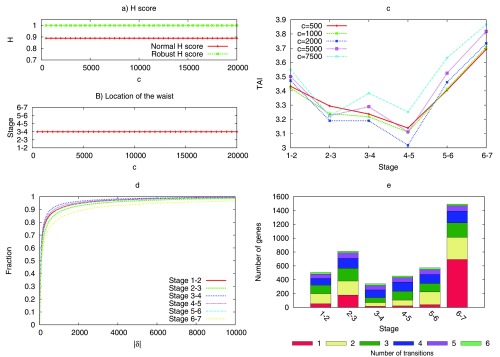
*Arabidopsis thaliana* results using absolute expression levels. Graph ( **a**) shows the hourglass score (normal and robust) as a function of the transition threshold *c*. Graph ( **b**) shows the location of the hourglass waist (stage-pair) as a function of the transition threshold *c*. Graph ( **c**) shows the Transcriptome Age Index of transitioning genes for five different values of *c* (chosen so that the number of genes with known age index assigned to each stage-pair is at least 170). Graph ( **d**) shows the CDFs of the expression level absolute variations | *δ*| across successive stage-pairs. Graph ( **e**) shows the transitioning genes with *c* = 5000. The transitioning genes constitute 8% of all genes in that dataset. 48% of those genes transition in a single stage-pair. Of the remaining, 38% transition only in consecutive stage-pairs. [Dataset 13].

## Note added in proof

Dedicated to the memory of the first author, Saamer Akhshabi, who passed away on March 6, 2014.
