# Supplementary material for: An explanatory evo-devo model for the developmental hourglass
Source: F1000Res. 2014 Dec 18;3:156. Originally published 2014 Jul 8. [Version 2] doi: 10.12688/f1000research.4583.2 (PMC4156030; doi:10.12688/f1000research.4583.2)
Supplement: Peer review history

# Peer review: Referee response for version 2

**DOI:** 10.5256/f1000research.6375.r7316

**Published:** 2015-01-14

**Authors:** Marcel Quint, Hajk-Georg Drost

**Affiliations:** 1 Department of Molecular Signal Processing, Leibniz Institute of Plant Biochemistry, Halle, Germany; 2 Institute of Computer Science, Martin Luther University Halle–Wittenberg, Halle, Germany

We highly appreciate that the authors carefully addressed all our concerns and we now have no further reservations.

 We also add a detailed response to the rebuttal letter:

  

1. **Point#1 of our summarized comments to the first version of the manuscript:** *“The authors do cite Raff in the first sentence of their introduction, but should state more clearly in which manner their model was inspired by Raff's hypothesis.”*
***Authors' response:*** **The reviewers are right to highlight the connection between our “increasing regulatory sp*ecificity” assumption and Raff’s hypothesis. We have included a paragraph in the Discussion section to put Raff’s hypothesis in the context of this work.*
*A first remark is that our assumption is consistent with Raff’s basic premise that modularity increases as the embryo develops. Second, Raff’s hypothesis (see chapter-6 of [1], and in particular Figures 6.6 and 6.7) also states that the “level of interaction” or the “interconnectivity between (body) elements” is maximized at the phylotypic stage. This may be viewed initially as a contradiction between Raff’s hypothesis and our increasing specificity assumption, which states that the density of regulatory interactions is maximized at the earliest stages of development. Note however that Raff’s hypothesis was not stated in terms of gene regulatory interactions -- he was referring more generally to “developmental flexibility”, arguing that early development is flexible because it governs robust and general global patterning processes, late development is also flexible because “signaling events within the primordia are little influenced by events in other primordia”, while mid-development (phylotypic stage)  is least flexible because of the “high interconnectivity between elements that will later come to represent separate modules.” If we think of “developmental flexibility” as the ability of an embryo to survive gene mutations and rewiring at different stages of the developmental process, Raff’s hypothesis is actually consistent with our results regarding the “lethality probability” at each developmental stage (see Figs 6-B, 7-B amd 8-B). The lethality probability is maximized at the phylotypic stage, and it is significantly lower at early and late developmental stages, following the same pattern with Raff’s “developmental flexibility”.*
**Our comment to authors' response: **We appreciate that the authors now differentiate between Raff’s hypothesis concerning the modularization (interconnectivity) between body elements (modules) and their "increasing regulatory specificity“ assumption. We agree that Raff’s hypothesis is consistent with the "lethality probability“ proposed by the authors in terms of the observed pattern of maximal interconnectivity and maximal probability of lethal effects during the phylotypic stage (mid embryogenesis). However, we would like to point out that a causal relationship between the observed patterns remains to be tested.
2. **Point#2 of our summarized comments to the first version of the manuscript:** *“Correlate and reference major assumptions of the DGEN model that seem plausible with findings from experimental studies that validate this plausibility.”*
**
***Authors' response to Point #2:  **First, to the extent of our knowledge there is no prior work that **directly **validates the assumption of increasing regulatory specificity as formulated in our paper (i.e., with the specificity of a developmental stage defined as the topological density of regulatory edges in that stage). We are planning to examine the validity of this assumption in the future using various approaches:*
**
* First we plan to analyze the aforementioned developmental GRNs for the sea-urchin that have been directly examined by Dr. Davidson’s lab at Caltech.*
**
* Second, we are in the process of examining other genomic metrics, which can be viewed as “proxies” to regulatory specificity. For example, we have defined new measures to quantify how specific a gene’s expression profile in a developmental stage is compared to other developmental stages, which we have tentatively named as “developmental stage specificity index (DSI)”.  Similarly, we defined “tissue specificity index (TSI)”, measuring the bias of a gene’s expression in a specific tissue compared to many other tissues. Analyses of these measures using genomic data from Drosophila so far have revealed generally increasing trends, which are consistent with the idea of increasing specificity. However we are still working on generalizing these analytical tools to data sets generated by different methods and from different species.  Importantly, our model focuses DGENs underlying development, and genomic data can obscure the true signals from genes constituting DGENs.  Thus we are also working on examining these specificity measures using inferred DGENs.  Additionally, we are developing other methods to approximate functional specificity. We hope to complete these analyses as a follow-up paper. The current paper provides theoretical motivation.*
**
* Third, as the reviewers also point out, there is a large body of prior work in developmental biology that confirms the increasing modularity in the developing embryo (for instance, see Wagner et al., 2007) as well as the increasing specificity of the developmental process at the signaling or genomic level. The connections between this pattern of increasing modularity and the structure of the underlying gene regulatory networks are still not well understood however. We think that it would be misleading if we had argued that these modularity patterns provide a direct validation for our “increasing regulatory specificity” assumption. Please note that we have cited the paper by Wagner et al. at the Introduction and Model Description sections, providing some connections between this work and earlier work on the role of modularity in developmental biology. *
**Our comment to authors' response:** The authors point out that "the connections between this pattern of increasing modularity and the structure of the underlying gene regulatory network are still not well understood“. This statement is valid for experimental studies that aim to address this presumptive causal relationship in a systematic manner and we appreciate that the authors share their future plans to introduce sufficient measures (DSI and TSI) to better understand the nature of this relationship.
3. **Point#3 of our summarized comments to the first version of the manuscript:** ****** *“Correlate the output of the DGEN simulation with existing findings of studies addressing similar questions of the causality of the developmental hourglass phenomenon and discuss potential simplifications of the model assumptions that do not fit experimental results or that are not derivable via experiments yet.”*
**
***Authors' response to Point #3:** This is a very interesting idea and we thank the reviewers for this suggestion. We are planning to pursue this investigation in a follow-up paper that will also include the previously mentioned analysis of sea-urchin GRNs as well as the DSI/TSI results. We believe that it would be distracting to try to include all these results in this first paper, given that the main focus of this work has been on the computational model and the computational results. We are also open to collaborate in this investigation with the reviewers or other researchers working in this area, if they are interested. *
**Our comment to authors' response:** ****We are delighted to hear that the authors address this question in a follow up paper. We gladly offer our expertise to collaboratively investigate the evolutionary process on the regulatory level that might have led to a correlation between the maximized evolutionary age of expressed genes during mid-embryogenesis and the interconnectivity of (body) modules that are hypothesized to cause the morphological patterns during the phylotypic stage.
4. ***Points#4 and 5 of our summarized comments to the first version of the manuscript: **Include a clear statement how the exact values of the stage specificity (regulatory specificity) s(l) have been derived.  State more clearly that s(l) is the crucial parameter determining the observed phenomena.”*
**
***Authors' response to Points #4 and 5: **The regulatory specificity functions we experimented with are arguably the simplest one could think of. Specifically, we start (Model-1) with the simplest hypothesis that the regulatory specificity does not vary across developmental stages (in the paper we show results for s=0.5 but we have generated results for all values 0.1, 0.2, ... 0.9 and the conclusion remains that when the specificity is constant the network does not evolve into an hourglass shape). Then, we consider (Model-2) a linearly increasing specificity function. We do not argue that this is realistic or that there are experimental results that suggest this linearity or even the increasing trend. It is just the simplest and most parsimonious model that leads to the emergence of an hourglass pattern. Finally, in Figures-9 and 10 we consider a more general specificity function that increases in a non-linear manner, providing a simple way to control the stage at which it gives the mid-range value 0.5. This allows us to examine how the shape of the specificity function s(l) affects the location of the hourglass waist. We hope that follow-up work will reveal experimentally the actual shape of the regulatory specificity function s(l) and there is no doubt that it will not be identical to any of these simple mathematical functions.*
**
* Please note that we have revised the paragraphs about “Model-1” and “Model-2” in the Simulation section to clarify these points. *
**Our comment to authors' response: **We thank the authors for clarifying the paragraphs about "Model-1“ and "Model-2“.
5. ***Specific reviewer comment on the first version of the manuscript **“For the probabilities P_{DL} and P_{DP} the explicit formula to compute the corresponding probabilities could also be included within the paragraph analogous to P_{RF}. This would enable a clearer reproducibility.“*
**
***Authors' response: **Actually there is no formula for the gene deletion and duplication probabilities. They are given probability values that do not depend on any other parameters. At each round of the simulation, a gene is duplicated with probability P_{DP}. If not rewired or duplicated, the gene is deleted with probability P_{DL}.  *
**Our comment to authors' response: **We thank the authors for clarifying this point for us.
6. ***Point#7 of our summarized comments to the first version of the manuscript: **“Explain why merging OrthoDB results with OrthoMCL results might be plausible and why comparing it with results obtained from phylostratigraphy is reliable.”*
**
***Authors' response to Point#7:** The phylostratigraphy technique as employed by Domazet-Loso et al. and Quint et al. involves the assignment of an evolutionary age to each gene in a given species, by tracing the most distant ancestral node containing at least one immediate daughter species with a detectable homologue. A phylogenetic tree of divergent species ranging from cellular organisms transitioning into simple and more complex eukaryota served as a reference to divide phyla radiating from consecutive ancestral points into distinct phylostratum layers. To determine the homologues, BLAST sequence similarity searches were done against complete genomes reliably annotated across the different phylostrata. Similarly, we obtained known homologues of each Drosophila gene from OrthoDB5 and OrthoMCL5 (that also use BLAST based searches) in the species depicted in Figure 4, and mapped them to six different “phylostrata” (marked in Figure 4). Akin to the method described in the above references, we assigned each gene to a specific “phylostratum” based on the farthest detectable homologue of that gene, and this is referred to as “age index” in the paper. This made it possible to compare the age index based results we obtain in Drosophila to the phylostratigraphy based assignments in Arabidopsis.*
**
* Homologues for Drosophila genes were sourced from these two databases, to increase coverage across the species depicted in the tree in Figure 4. Since these two databases use slightly different parameters to identify orthologs, we examined whether the usage of specific databases introduce bias by analyzing data from each database separately and observed that age index of each gene remained the same. *
**Our comment to authors' response: **We are glad that the authors carefully addressed our initial concerns about the reliability of results obtained from merging methods of gene homology detection. Since the observed age indices are not influenced by the database used to assign gene homology relationships, we agree that merging OrthoDB5 and OrthoMCL5 results do not bias this specific analysis.

 

**Reviewer summary**

 All remaining concerns were carefully revised and we thank the authors for investing the additional time to respond to our review in such detail.

We have read this submission. We believe that we have an appropriate level of expertise to confirm that it is of an acceptable scientific standard.

# Peer review: Referee response for version 2

**DOI:** 10.5256/f1000research.6375.r7095

**Published:** 2014-12-29

**Authors:** Gourab Ghoshal

**Affiliations:** 1 Department of Earth and Planetary Sciences, Harvard University, Cambridge, MA, USA

The authors have addressed all my concerns appropriately. I approve this manuscript in its current form with no reservations.

I have read this submission. I believe that I have an appropriate level of expertise to confirm that it is of an acceptable scientific standard.

# Peer review: Referee response for version 1

**DOI:** 10.5256/f1000research.4902.r5391

**Published:** 2014-08-06

**Authors:** Gourab Ghoshal

**Affiliations:** 1 Department of Earth and Planetary Sciences, Harvard University, Cambridge, MA, USA

The manuscript by Akhshabi *et al.* proposes and describes in detail an evolutionary model that qualitatively reproduces the "Hourglass Effect"---a phenomenon (in the biological context) whereby there is increased morphological divergence at early and late stages of embryonic development separated by increased conservation in the phylotypic stage. 

The proposed model is based on a hierarchical network representation of the Gene Regulatory Network - the so-called Developmental Gene Execution Network (DGEN) - and evolution proceeds through a series of stochastic perturbations involving gene duplication, re-wiring and deletion. The manuscript suggests that the key factor that reproduces the hourglass effect is the monotonicity in "time" (increasing) of a function they call specificity s(i) (a measure of likelihood of a gene regulating descendant genes at later times) that competes against perturbative effects such that there is a "waist" of the number of transitions genes w(l) at intermediate times. They then test predictions of the model in two datasets of developmental data *Drosophilia melanogaster* and *Arabidopsis Thaliana,* finding good qualitative agreement. 

The manuscript is properly motivated and well written (with some caveats, see comments below). The model proposed is quite intuitive and compelling and represents an excellent first attempt in beginning to uncover the mechanism behind this intriguing phenomenon. I anticipate that this will have high impact (considering the hourglass effect transcends biological phenomena and is also found in "designed" systems such as the Internet *protocol stack*) in multiple fields. Consequently I strongly endorse this manuscript. 

I do however have some concerns about some of the details about the model and therefore propose the following that may improve the readability of the manuscript and the plausibility and robustness of the model:

1. The section describing the model is a bit dense, and can benefit from some rewriting. For example it is a bit hard to follow what the significance of a spatial domain is and its relevance to the model. In addition a bewildering plethora of parameters are introduced, that can be a bit overwhelming for the reader. Consequently I suggest putting all the important and relevant parameters in a table for easy access to the reader. The section on Rewiring (RW) is quite hard to read and will benefit from the introduction of explicit equations similar to that for P_{RF}. 
2. Which brings me to my second point about the assumptions of the model. It seems to me that two key factors lead to the hourglass effect: a) the montonocity of s(l) with stage l and likewise b) of P_{RF} with r. While the choice of this dependence for the specificity  is a simple linear dependence (which makes sense as a first pass), the choice of function for P_{RF} is highly non-trivial. Why not for example choose a simple linear dependence such as P_{RF} ~ r? The reason why I say this is that the authors would like to propose the most general and minimal model to explain this phenomenon. And it is not clear to me whether the specific choice of the functional dependences matter. For example with a non-linear choice of P_{RF} we see that a non-linear s(l) has the effect of shifting the waist. But what about a linear choice of P_{RF} and s(l)? Does that still preserve the hourglass effect? In my opinion a truly robust model will state that the main things that determine the hourglass are the monotonic dependencies in combination with non-linear or linear choices of the functions independent of their specific forms. This will allow the model to be applied to multiple settings.
  At the moment I'm a bit concerned that the non-trivial choice of P_{RF}, as it stands in the manuscript, might be key to the observed effect. If it is indeed so, then one must motivate why one must make this particular choice. Same goes for the slightly peculiar choice for the non-linearity of s(l) introduced later in the model. The authors will do well to explain in detail what motivated them to make such a choice. 
  Additionally it would be helpful if the authors make it clearer (than they already have) that effects such as duplication and deletion are there only to make the model more realistic in terms of maintaining the number density of genes across different stages and play no part in the hourglass effect (or at least that's how I understand it).
3. Some minor nitpicks:
  ​
  1. In the section Model Description, the authors refer to the Wright-Fisher model. I was not aware of what this was and had to look it up, so a reference for the reader should be provided.
  2. The Hourglass score H is determined through Mann-Kendall statistics. A brief description of the method should be provided for the benefit of the reader (maybe in the supplementary material). Additionally, why this particular choice? Presumably any statistical test for monotonic trends should be robust to the parameters of the model. Would there be much of a difference is one used the Spearman rho for example?
  3. Finally I think the authors will do well to highlight the importance of Specificity right at the outset of the manuscript and move some simplified variant of the segment "What is the reason behind the hourglass shape of DGENS?" (page 7, line 11, second column) to somewhere in the introduction.

I have read this submission. I believe that I have an appropriate level of expertise to confirm that it is of an acceptable scientific standard, however I have significant reservations, as outlined above.

# Response

**Published:** 2014-12-11

**Authors:** Constantine Dovrolis

**Affiliations:** Georgia Institute of Technology, USA

First, we would like to thank the author of this review for his very thorough reading of our paper. His comments and suggestions have been extremely helpful in revising the paper (please look at Version-2). We would also like to apologize for not revising the paper earlier.

- **Review**: *“The section describing the model is a bit dense, and can benefit from some rewriting. For example it is a bit hard to follow what the significance of a spatial domain is and its relevance to the model.”*
**Response**: We hope our extensive revision has mitigated this problem of the previous version of the paper.
  We explain what we mean by “spatial domains” early in the paper (and in Fig-1) because it is an important and necessary concept when we justify later in the paper the assumption of increasing regulatory specificity (see paragraph that starts with “The major assumption is that the regulatory specificity…”). It is the formation of those distinct spatial domains (or “ modules”) that explains why the regulatory specificity is probably decreasing as development progresses.
- **Review**: *“The section on Rewiring (RW) is quite hard to read and will benefit from the introduction of explicit equations similar to that for P_{RF}."*
**Response**: We have revised that paragraph to make it more clear.
- **Review**: *It seems to me that two key factors lead to the hourglass effect: a) the monotonicity of s(l) with stage l and likewise b) of P_{RF} with r. While the choice of this dependence for the specificity  is a simple linear dependence (which makes sense as a first pass), the choice of function for P_{RF} is highly non-trivial. Why not for example choose a simple linear dependence such as P_{RF} ~ r? For example with a non-linear choice of P_{RF} we see that a non-linear s(l) has the effect of shifting the waist. But what about a linear choice of P_{RF} and s(l)? Does that still preserve the hourglass effect? In my opinion a truly robust model will state that the main things that determine the hourglass are the monotonic dependencies in combination with non-linear or linear choices of the functions independent of their specific forms.”*
**Response**: We chose the non-linear P_{RF} function shown in Fig-2 because it is quite flexible and it allows us to examine how the shape of this curve affects the behavior of the model by simply varying the parameter z. Please note that when z is equal to one this function is almost linear. As discussed in Fig-10 however, as z decreases towards one the location of the hourglass waist also decreases. Consequently, we expect that a linearly increasing P_{RF} function would result in a shape that resembles a “funnel” rather than an hourglass.
  We hope that new experimental work will provide some direct evidence for the shape of this function in the near future.
***Review**: “In the section Model Description, the authors refer to the Wright-Fisher model. I was not aware of what this was and had to look it up, so a reference for the reader should be provided.”*
- **Response**: We have added a reference for this model:
  “Ewens WJ. Mathematical Population Genetics. Springer-Verlag Berlin New York. 1979. Reference Source”
- **Review**: *“The Hourglass score H is determined through Mann-Kendall statistics. A brief description of the method should be provided for the benefit of the reader (maybe in the supplementary material). Additionally, why this particular choice? Presumably any statistical test for monotonic trends should be robust to the parameters of the model. Would there be much of a difference is one used the Spearman rho for example?”*
**Response**: We have added a reference for this statistic:
  "Gibbons JD, Chakraborti S: Nonparametric Statistical Inference. Marcel Dekker New York. 2003. Reference Source”
  The Mann-Kendall statistical test is one of the most widely used statistics to assess the significance of trends in data (Gibbons and Chakraborti 2003). Mann-Kendall and Spearman’s tests perform highly similar in data analyses (e.g., Yue *et al*., 2002; Shadmani *et al.*, 2012).
- **Review**: “ *Finally I think the authors will do well to highlight the importance of Specificity right at the outset of the manuscript and move some simplified variant of the segment "What is the reason behind the hourglass shape of DGENS?" (page 7, line 11, second column) to somewhere in the introduction.”*
**Response**: The reviewer is right. We revised the fourth paragraph of the introduction to include a “hint” about the main reason behind the hourglass effect. Obviously, we cannot write much more at that early point of the paper because we still haven’t defined what we mean by DGEN, RFs, cascades, etc.

# Peer review: Referee response for version 1

**DOI:** 10.5256/f1000research.4902.r5650

**Published:** 2014-07-31

**Authors:** Dmitri Krioukov

**Affiliations:** 1 Department of Physics, Northeastern University, Boston, MA, USA

The article proposes and analyzes a network model that is capable of reproducing the hourglass effect in the number of genes that undergo a functional state transition as a function of the developmental stage. The hourglass effect refers to higher morphological divergence at earlier or later stages of embryonic developmental, compared to medium stages. The main idea of the model is to explain the waist of this hourglass (the number of "transitioning genes" w(l) having a minimum at medium developmental stages l) by an interplay between the impact of random rewiring events in the developmental gene execution network (DGEN) and stage specificity. The DGEN is a network representing regulatory interactions between genes at different developmental stages, while stage specificity s(l) defines the probability 1-s(l) with which a gene at stage l regulates a gene at stage l+1. One of the key assumptions in the model is that s(l) is an increasing function of l. As a result of the interplay between a decreasing impact of DGEN perturbations as a function of l, and increasing s(l), the lethality of perturbations is maximized at mid l's, leading to evolutionary incentives to minimize w(l) at those mid stages. The article consists of three parts. The first part describes the model in detail. The second part discusses extensive simulation results of the model. The third part presents an extensive analysis of existing developmental data on *Drosophila melanogaster *and *Arabidopsis thaliana* in the model context.

The most interesting aspect of the article is that provides a possible intriguing explanation of the hourglass effect in developmental biology, which may foster future creative thinking and research in this important direction. The most obvious reservation one can express about the article is that the model cannot be currently refuted since it is formulated at the DGEN level, and there is currently no data from which a reliable DGEN reconstruction would be possible. The fact that some outcomes of model simulations qualitatively agree with available data does not directly validate either the model, or its main assumptions. The article does not contain such claims, however, that would not be supported by the data.

My minor comments that may help to improve the article are:

1. The main idea of the model is buried somewhere on page 6 in the middle of a paragraph after sentence "What is the reason behind the hourglass shape of DGENs?" Why not explain this as early as possible, certainly in the Introduction, if not in the abstract?
2. The third paragraph in the Introduction reads somewhat unclear and imprecise, as well as the "Developmental gene execution networks" section.
3. When D(g) and Gamma notations first appear, it's not spelled out what they are.
4. DGEN removals upon DF events have a clear meaning. But what do replacements correspond to in reality?
5. The Mann-Kendall statistics could be briefly described, or at least a reference could be provided.

I have read this submission. I believe that I have an appropriate level of expertise to confirm that it is of an acceptable scientific standard.

# Response

**Published:** 2014-12-11

**Authors:** Constantine Dovrolis

**Affiliations:** Georgia Institute of Technology, USA

First, we would like to thank the author of this review for his thorough reading of our work. His comments have been very helpful in revising this paper (please see Version 2). We would also like to apologize for not revising the paper earlier.

- **Review**: *“The most obvious reservation one can express about the article is that the model cannot be currently refuted since it is formulated at the DGEN level, and there is currently no data from which a reliable DGEN reconstruction would be possible. The fact that some outcomes of model simulations qualitatively agree with available data does not directly validate either the model, or its main assumptions. The article does not contain such claims, however, that would not be supported by the data.”*
**Response**: It is true that a direct validation of the model is not possible with any currently available data (at least to the extent of our knowledge). Such a direct validation would require the reconstruction of the complete DGEN for specific model organisms, i.e., knowing not only the expression profile of each gene as a function of developmental time and at different tissues of the embryo but also knowing the regulatory inputs for each gene during development. However, there are research groups that are working in that direction (e.g., Prof. Davidson’s group at Caltech is gradually “reverse-engineering” the DGEN of the sea-urchin). Our hope is that a direct validation of our model will be possible within the next few years.
  Having said that however, even though our model cannot be directly validated, we are providing indirect evidence that certain predictions of the model are true in Drosophila and Arabidopsis (see Results - Data Analysis section). Consequently, the model should not be viewed as completely hypothetical either.
- **Review**: *“The main idea of the model is buried somewhere on page 6 in the middle of a paragraph after sentence "What is the reason behind the hourglass shape of DGENs?" Why not explain this as early as possible, certainly in the Introduction, if not in the abstract?”*
**Response**: The reviewer is right. We revised the fourth paragraph of the introduction to include a “hint” about the main reason behind the hourglass effect. Obviously, we cannot write much more at that early point of the paper because we still haven’t defined what we mean by DGEN, RFs, cascades, etc. The revised paragraph is:
  “The model predicts that the evolutionary process shapes the DGENs of a population in the form of an hourglass, under fairly general assumptions. Specifically, the number of genes at each developmental stage follows an hourglass pattern, with the smallest number at the “waist” of the hourglass. The main condition for the appearance of the hourglass pattern is that the DGEN should gradually get sparser as development progresses, with general-purpose regulatory genes at the earlier developmental stages and highly specialized regulatory genes at the later stages. Under this assumption, the model predicts that gene regulatory changes  or rewiring in mid-development are more likely to cause cascades of removing non-essential genes from the DGEN, compared to early or late developmental stages. Another model prediction is that the evolutionary age of DGEN genes also follows an hourglass pattern, with the oldest genes concentrated at the waist.”
- **Review**: “ *When D(g) and Gamma notations first appear, it's not spelled out what they are.”*
**Response**: We checked this. The notation D(g) is introduced at the end of the “Developmental gene execution networks” section, and that is the first time it appears in the paper. The notation Gamma first appears in the “Developmental Failure (DF)” paragraph, and that is also where it is defined.
- **Review**: *“DGEN removals upon DF events have a clear meaning. But what do replacements correspond to in reality?”*
**Response**: DGEN replacements model the effect of selection: an embryo that did not develop properly (DF) dies and its DGEN is removed from the population, while healthy embryos develop and eventually give birth to new a embryo with the same genotype as its parent (asexual reproduction).
- **Review**: *“The Mann-Kendall statistics could be briefly described, or at least a reference could be provided.”*
**Response**: We have added a reference for this statistic:
  "Gibbons JD, Chakraborti S: Nonparametric Statistical Inference. Marcel Dekker New York. 2003. Reference Source”

# Peer review: Referee response for version 1

**DOI:** 10.5256/f1000research.4902.r5563

**Published:** 2014-07-24

**Authors:** Marcel Quint, Hajk-Georg Drost

**Affiliations:** 1 Department of Molecular Signal Processing, Leibniz Institute of Plant Biochemistry, Halle, Germany; 2 Institute of Computer Science, Martin Luther University Halle–Wittenberg, Halle, Germany

This study by Akhshabi *et al*. aims to provide the community with an explanatory theoretical model for the developmental hourglass phenomenon. Motivated by the question concerning the regulatory mechanism causing the developmental hourglass pattern during embryogenesis, the authors developed an evolutionary model of regulatory gene interactions during development to identify the conditions under which the molecular hourglass effect might emerge in general.

For this purpose their evolutionary model focuses on hierarchical gene regulatory networks that control the corresponding developmental processes. Based on the output of the model the authors predict the emergence of an hourglass pattern in the structure of a temporal representation of the underlying gene regulatory network and correlate this effect with the evolution of protocol architecture found for the Internet’s “protocol stack”.

Based on this universal finding the authors speculate that the developmental hourglass pattern might be a causal effect of a common organization principle during the establishment of complexity and is based on the underlying hierarchical network structure.

**The paper is very interesting, well written, and will potentially help the community to better understand the developmental hourglass phenomenon. However, we see some issues with the model assumptions and strongly suggest including the following aspects in a revised version.**

Summary of the points that have to be addressed: 

1. Include a clear reference and paragraph to Raff's hypothesis and how it influenced the modeling of DGEN.
2. Correlate and reference major assumptions of the DGEN model that seem plausible with findings from experimental studies that validate this plausibility.
3. Correlate the output of the DGEN simulation with existing findings of studies addressing similar questions of the causality of the developmental hourglass phenomenon and discuss potential simplifications of the model assumptions that do not fit experimental results or that are not derivable via experiments yet.
4. Include a clear statement how the exact values of the stage specificity (regulatory specificity) *s(l)* have been derived.
5. State more clearly that *s(l)* is the crucial parameter determining the observed phenomena.
6. Correct the TAI formula.
7. Explain why merging OrthoDB results with OrthoMCL results might be plausible and why comparing it with results obtained from phylostratigraphy is reliable.
8. Discuss more clearly how the DGEN model can help to find causal processes of the developmental hourglass phenomenon based on recent knowledge: the three predictions of the DGEN model a) – c) mentioned in the Discussion section seem not integrative enough to allow future studies to correlate existing results provided by the community with the predictions obtained by the DGEN model.

Specific comments to the separate sections:

In general, most of our criticisms concern the model description and the methods section. Both are integral to the validity of the obtained results, which are nicely reported and discussed. 

**Introduction** 

Modeling the evolution of embryonic development as directed acyclic graph (DAG) in which the nodes correspond to state-transitioning genes and the edges model directed regulatory interactions causing significant activity change at the corresponding stage seems plausible. The authors refer to this model as DGEN. A testable hypothesis proposed by the authors is that if regulatory genes become increasingly function-specific as development progresses, the network gradually should become sparser in that direction and evolutionary older genes should be concentrated at the waist of the hourglass (Figure 1). 

This hypothesis is essential to detect possible regulatory mechanisms that cause the developmental hourglass pattern. We therefore suggest including references to existing biological studies investigating the phenomenon of morphological complexity being correlated with regulatory specialization to support the validity and plausibility of their DGEN model assumptions. 

A biological motivation of this hypothesis comes from Rudolf A. Raff who proposed that early embryogenesis is governed by global pattern formation processes and that during later development, the embryo is being organized into developmental modules, allowing each module to differentiate autonomously. Raff furthermore proposed that the phylotypic stage marks the transition from early global development to the modular mode later in development ^1^. 

The authors do cite Raff in the first sentence of their introduction, but should state more clearly in which manner their model was inspired by Raff's hypothesis. 

Numerous studies investigated the phenomenon that increasing morphological complexity is correlated with increasing modularity and specialization of the underlying regulatory apparatus ^2^. Here the authors predict that the main condition leading to the appearance of the hourglass pattern is that the DGEN should gradually get sparser as development progresses, with general-purpose regulatory genes at the earlier developmental stages and highly specialized regulatory genes at the later stages, expressing the oldest genes during mid development. 

We suggest to also include findings investigating the functionality and age of the genes that have been shown to be active during mid embryogenesis to correlate potential outcomes of the DGEN prediction that evolutionary older genes should be concentrated at the waist of the hourglass with results obtained by recent studies ^3^ ^4^ ^5^ ^6^ ^7^ ^8^ ^9^ ^10^ ^11^ ^12^. These aforementioned studies were able to show that during mid embryogenesis evolutionary old genes or evolutionary old processes that are shared within and between phyla are most active. 

In our opinion it would be very interesting to test the degree of intersection between genes predicted to be causal for the waist of the DGEN hourglass and evolutionary conserved genes that have been found to be expressed during mid embryogenesis. We are aware that the model uses the number of genes at each developmental stage combined with the age of the corresponding genes to observe constraints during development. Hence, only the authors can estimate how much effort would be needed to identify such intersecting genes. 

If the search for intersecting genes returned by the DGEN model during mid development and genes found by previous studies should be possible, the authors could integrate a motivation section to find these intersecting genes within the Introduction section.  

**Model description** 

The model description is quite intuitive. However, as the validity of the whole study depends on the model parameters chosen, several points need clarification including the rationale and motivation to choose exactly these parameters. 

One of the most crucial parameters of the model is the regulatory specificity *s(l)*, with 0 <= *s(l)* <= 1. We think, that adding a more detailed paragraph discussing how *s(l)* is being estimated, would be highly beneficial due to the importance for the parameter *s(l)* for the entire model and predictions. In Model-1-4 the authors choose three different values for *s(l)*: In Model-1: *s(l)* = 0.5 and in Model-2-4: *s(l)* = l/L, and in Figure 9 a non-linear function *s(l)* = 0.9 – (0.8 / 1 + exp(\gamma - l) ). A gene g at stage l is modeled to act as upstream regulator for a gene g’ at stage l + 1 with probability *s’(l)* = 1 – *s(l)*. From this follows that each potential upstream regulator in stage l has the same probability *s’(l)* to regulate genes g’ in stage l + 1. In other words, Model-1 assigns each potential upstream regulator with the same probability *s’(l)* = 1 – 0.5 = 0.5 to regulate genes g’ in stage l + 1, in Model-2: *s*’(l) = 1 – l/L = const., and for the non-linear function *s(l)*: *s’(l)* = 1 – (0.9 – (0.8 / 1 + exp(\gamma - l) )) = const. 

It would be advantageous, if the authors could include motivations for the three cut-offs, especially how they derived the sigmoid-like mathematical function (Figure 10, legend caption) they later denote as non-linear function *s(l)*. We do see that modeling the stage specificity with a constant value, an increasing value, and a non-linear function is plausible, but a clear motivation or derivation of the exact values would be beneficial for a better understanding of the modeling process. 

For the probabilities *P _DL_* and *P _DP_* the explicit formula to compute the corresponding probabilities could also be included within the paragraph analogous to P _RF_. This would enable a clearer reproducibility. 

The authors discuss that their major assumption is that the regulatory specificity increases substantially as development progresses, hence the DGEN becomes gradually sparser along the developmental time axis starting with *s(1)* ~ 0, … , *s(L)* ~ 1. Here the authors should include references to experimental observations that support their assumption. Since Models 2-4 rely on this assumption, published experimental studies in line with this assumption need to be referenced. Otherwise, the models applied would seem largely academic.  

**Methods** 

Hourglass score H

Here *w(l)* is defined as the number of transitioning genes in stage l. In Figure 3 and in section *'Hourglass shape'* *w(l)* is referred to as *stage width*. In case the definition of *w(l)* is equivalent to the *stage width*, it would be beneficial for the reader to see the term *stage width* in the same sentence as the initial definition of *w(l)*. A correct understanding of the *stage width* is important to understand the measurement of *H*.

Furthermore, the univariate Mann-Kendall statistic should be referenced to allow a non-statistical community to better understand this way of statistical modeling. 

Transcriptome age index (TAI)

In this section the authors describe and perform two different methods to assign each gene a corresponding evolutionary age. For *D. melanogaster* they collected groups of orthologs from OrthoDB and OrthoMCL, whereas for *A. thaliana* they obtained phylostratum assignments from Quint et al. ^10^ that was derived by phylostratigraphy[ref-13.]We do not understand the motivation of merging orthologous gene groups obtained from OrthoDB and OrthoMCL, as both approaches apply different parameters and heuristics to determine orthologous genes. Was this done to maximize the number of potential orthologs to be included in the study? In addition, why did the authors use a different approach for the plant gene set? This seems somewhat inconsistent and statement concerning the plausibility to compare results returned by the DGEN model based on age assignments obtained from phylostratigraphy ( *A. thaliana*) with OrthoDB and OrthoMCL merged orthologs ( *D. melanogaster*) would be appreciated. 

Furthermore, the database specific parameters they used in OrthoDB and OrthoMCL should be mentioned as well as the database version they used, e.g. OrthoDB2, or OrthoDB3, … , or OrthoDB6. 

Age index for each stage-pair

There seems to be a typo within the TAI formula. As defined by Domazet-Loso and Tautz ^6^ and adapted to the author’s notation the formula of the TAI should begin *TAI(l)* not *TAI(1).* 

**Results** 

The authors show based on their findings returned by Model-2-4 that the increasing specificity assumption is the key property behind the developmental hourglass effect. We think that this statement is not clear enough. The authors could phrase more clearly what they mean by “ *key property behind the hourglass effect”* inferred from Model-2-4. 

Age of genes

Here the authors motivate that defining the age of a gene as *A(g) = i – t _0_(g)* is plausible, because a rewiring event may give a gene a new function, at least in terms of its upstream and downstream regulators. This statement needs validation from the literature and from experimental studies that already tested this hypothesis for specific rewiring events. 

Furthermore, the authors show (Figure 8c) that the evolutionary age at stage l follows the same pattern as the lethality probability: gradually increasing until the waist of the hourglass, and then gradually decreasing. This finding should be correlated with the findings by Galis and Metz ^3^ and should be discussed more clearly in the Discussion. 

In the first sentence of the last paragraph before the Discussion, "… *and TAI profiles from Arabidopsis" *should be referenced to reference 11 and not 12. 

**Discussion** 

The authors discuss that the observed pattern of conservation (developmental hourglass) may stem from fundamental organization principles and that the exact origin of these principles remains elusive. Here the authors should include existing studies that also aim at predicting a universal organization principle during development (modularization, etc.) and what exact problems are preventing studies to elucidate this universal phenomenon. We do note, that the authors cite studies that aim to explain the effects causing the early phase of the developmental hourglass ^14^ ^15^ ^16^ and studies aiming to explain effects causing the late phase of the developmental hourglass ^1^, but we think that it should be explicitly stated that the model is designed to test aspects of Raff’s initial hypothesis and that also more recent studies have been investigating the underlying reasons for the pattern in the early phase of the hourglass ^17^.

We have read this submission. We believe that we have an appropriate level of expertise to confirm that it is of an acceptable scientific standard, however we have significant reservations, as outlined above.

## References

[1] RaffRA: The Shape of Life: Genes, Development and the Evolution of Animal Form. *(Univ. Chicago Press)*.1996;

[2] WagnerGPPavlicevMCheverudJM: The road to modularity. *Nature Reviews Genetics*.2007;8(12) : 10.1038/nrg2267 921-931 10.1038/nrg2267 18007649

[3] GalisFMetzJA: Testing the vulnerability of the phylotypic stage: on modularity and evolutionary conservation. *J Exp Zool.*.2001;291(2) :195-204 1147991810.1002/jez.1069

[4] Hazkani-CovoEWoolDGraurD: In search of the vertebrate phylotypic stage: a molecular examination of the developmental hourglass model and von Baer's third law. *J Exp Zool B Mol Dev Evol.*.2005;304(2) : 10.1002/jez.b.21033 150-158 10.1002/jez.b.21033 15779077

[5] RouxJRobinson-RechaviM: Developmental constraints on vertebrate genome evolution. *PLoS Genet.*.2008;4(12) : 10.1371/journal.pgen.1000311 e1000311 10.1371/journal.pgen.1000311 19096706PMC2600815

[6] Domazet-LošoTTautzD: A phylogenetically based transcriptome age index mirrors ontogenetic divergence patterns. *Nature.*.2010;468(7325) : 10.1038/nature09632 815-818 10.1038/nature09632 21150997

[7] KalinkaATVargaKMGerrardDTPreibischSCorcoranDLJarrellsJOhlerUBergmanCMTomancakP: Gene expression divergence recapitulates the developmental hourglass model. *Nature.*.2010;468(7325) : 10.1038/nature09634 811-814 10.1038/nature09634 21150996

[8] IrieNKurataniS: Comparative transcriptome analysis reveals vertebrate phylotypic period during organogenesis. *Nat Commun.*.2011;2(248) : 10.1038/ncomms1248 10.1038/ncomms1248 21427719PMC3109953

[9] LevinMHashimshonyTWagnerFYanaiI: Developmental milestones punctuate gene expression in the Caenorhabditis embryo. *Dev Cell.*.2012;22(5) : 10.1016/j.devcel.2012.04.004 1101-1108 10.1016/j.devcel.2012.04.004 22560298

[10] QuintMDrostHGGabelAUllrichKKBönnMGrosseI: A transcriptomic hourglass in plant embryogenesis. *Nature*.2012;490(7418) : 10.1038/nature11394 98-101 10.1038/nature11394 22951968

[11] SchepANAdryanB: A comparative analysis of transcription factor expression during metazoan embryonic development. *PLoS One.*.2013;8(6) : 10.1371/journal.pone.0066826 e66826 10.1371/journal.pone.0066826 23799133PMC3682979

[12] PiaseckaBLichockiPMorettiSBergmannSRobinson-RechaviM: The hourglass and the early conservation models--co-existing patterns of developmental constraints in vertebrates. *PLoS Genet.*.2013;9(4) : 10.1371/journal.pgen.1003476 e1003476 10.1371/journal.pgen.1003476 23637639PMC3636041

[13] Domazet-LosoTBrajkovićJTautzD: A phylostratigraphy approach to uncover the genomic history of major adaptations in metazoan lineages. *Trends Genet.*.2007;23(11) : 10.1016/j.tig.2007.08.014 533-539 10.1016/j.tig.2007.08.014 18029048

[14] RiedlRJefferiesRP: Order in living organisms: a systems analysis of evolution. *Wiley New York*.1978;

[15] ArthurW: Mechanisms of morphological evolution: a combined genetic, developmental, and ecological approach. *Wiley New York*.1984;

[16] RasmussenN: A new model of developmental constraints as applied to the Drosophila system. *J Theor Biol.*.1987;127(3) :271-299 343112610.1016/s0022-5193(87)80107-8

[17] KalinkaATTomancakP: The evolution of early animal embryos: conservation or divergence?. *Trends Ecol Evol.*.2012;27(7) :385-393 2252086810.1016/j.tree.2012.03.007

# Response

**Published:** 2014-12-11

**Authors:** Constantine Dovrolis

**Affiliations:** Georgia Institute of Technology, USA

First, we would like to thank the two authors of this review for their very thorough reading of our paper. Their comments and suggestions have been extremely helpful in revising the paper (please look at Version-2). We would also like to apologize for not revising the paper earlier.

- **Review**: *“Modeling the evolution of embryonic development as directed acyclic graph (DAG) in which the nodes correspond to state-transitioning genes and the edges model directed regulatory interactions causing significant activity change at the corresponding stage seems plausible. The authors refer to this model as DGEN. A testable hypothesis proposed by the authors is that if regulatory genes become increasingly function-specific as development progresses, the network gradually should become sparser in that direction and evolutionary older genes should be concentrated at the waist of the hourglass (Figure 1). This hypothesis is essential to detect possible regulatory mechanisms that cause the developmental hourglass pattern. We therefore suggest including references to existing biological studies investigating the phenomenon of morphological complexity being correlated with regulatory specialization to support the validity and plausibility of their DGEN model assumptions. A biological motivation of this hypothesis comes from Rudolf A. Raff who proposed that early embryogenesis is governed by global pattern formation processes and that during later development, the embryo is being organized into developmental modules, allowing each module to differentiate autonomously. Raff furthermore proposed that the phylotypic stage marks the transition from early global development to the modular mode later in development.”*
**Summary - Point#1:** *“The authors do cite Raff in the first sentence of their introduction, but should state more clearly in which manner their model was inspired by Raff's hypothesis.”*
**Summary - Point#2:** *“Correlate and reference major assumptions of the DGEN model that seem plausible with findings from experimental studies that validate this plausibility.”*
**Response to Point #1: **The reviewers are right to highlight the connection between our “increasing regulatory specificity” assumption and Raff’s hypothesis. We have included a paragraph in the Discussion section to put Raff’s hypothesis in the context of this work.
  A first remark is that our assumption is consistent with Raff’s basic premise that modularity increases as the embryo develops. Second, Raff’s hypothesis (see chapter-6 of [1], and in particular Figures 6.6 and 6.7) also states that the “level of interaction” or the “interconnectivity between (body) elements” is maximized at the phylotypic stage. This may be viewed initially as a contradiction between Raff’s hypothesis and our increasing specificity assumption, which states that the density of regulatory interactions is maximized at the earliest stages of development. Note however that Raff’s hypothesis was not stated in terms of gene regulatory interactions -- he was referring more generally to “developmental flexibility”, arguing that early development is flexible because it governs robust and general global patterning processes, late development is also flexible because “signaling events within the primordia are little influenced by events in other primordia”, while mid-development (phylotypic stage)  is least flexible because of the “high interconnectivity between elements that will later come to represent separate modules.” If we think of “developmental flexibility” as the ability of an embryo to survive gene mutations and rewiring at different stages of the developmental process, Raff’s hypothesis is actually consistent with our results regarding the “lethality probability” at each developmental stage (see Figs 6-B, 7-B amd 8-B). The lethality probability is maximized at the phylotypic stage, and it is significantly lower at early and late developmental stages, following the same pattern with Raff’s “developmental flexibility”.
**Response to Point #2:  **First, to the extent of our knowledge there is no prior work that **directly** validates the assumption of increasing regulatory specificity as formulated in our paper (i.e., with the specificity of a developmental stage defined as the topological density of regulatory edges in that stage). We are planning to examine the validity of this assumption in the future using various approaches:
  First we plan to analyze the aforementioned developmental GRNs for the sea-urchin that have been directly examined by Dr. Davidson’s lab at Caltech.
  Second, we are in the process of examining other genomic metrics, which can be viewed as “proxies” to regulatory specificity. For example, we have defined new measures to quantify how specific a gene’s expression profile in a developmental stage is compared to other developmental stages, which we have tentatively named as “developmental stage specificity index (DSI)”.  Similarly, we defined “tissue specificity index (TSI)”, measuring the bias of a gene’s expression in a specific tissue compared to many other tissues. Analyses of these measures using genomic data from Drosophila so far have revealed generally increasing trends, which are consistent with the idea of increasing specificity. However we are still working on generalizing these analytical tools to data sets generated by different methods and from different species.  Importantly, our model focuses DGENs underlying development, and genomic data can obscure the true signals from genes constituting DGENs.  Thus we are also working on examining these specificity measures using inferred DGENs.  Additionally, we are developing other methods to approximate functional specificity. We hope to complete these analyses as a follow-up paper. The current paper provides theoretical motivation.
  Third, as the reviewers also point out, there is a large body of prior work in developmental biology that confirms the increasing modularity in the developing embryo (for instance, see Wagner *et al*., 2007) as well as the increasing specificity of the developmental process at the signaling or genomic level. The connections between this pattern of increasing modularity and the structure of the underlying gene regulatory networks are still not well understood however. We think that it would be misleading if we had argued that these modularity patterns provide a direct validation for our “increasing regulatory specificity” assumption. Please note that we have cited the paper by Wagner et al. at the Introduction and Model Description sections, providing some connections between this work and earlier work on the role of modularity in developmental biology.
- **Review** *: “Numerous studies investigated the phenomenon that increasing morphological complexity is correlated with increasing modularity and specialization of the underlying regulatory apparatus ^2^. Here the authors predict that the main condition leading to the appearance of the hourglass pattern is that the DGEN should gradually get sparser as development progresses, with general-purpose regulatory genes at the earlier developmental stages and highly specialized regulatory genes at the later stages, expressing the oldest genes during mid development. We suggest to also include findings investigating the functionality and age of the genes that have been shown to be active during mid embryogenesis to correlate potential outcomes of the DGEN prediction that evolutionary older genes should be concentrated at the waist of the hourglass with results obtained by recent studies ^3 4 5 6 7 8 9 10 11 12^. These aforementioned studies were able to show that during mid embryogenesis evolutionary old genes or evolutionary old processes that are shared within and between phyla are most active. In our opinion it would be very interesting to test the degree of intersection between genes predicted to be causal for the waist of the DGEN hourglass and evolutionary conserved genes that have been found to be expressed during mid embryogenesis. We are aware that the model uses the number of genes at each developmental stage combined with the age of the corresponding genes to observe constraints during development. Hence, only the authors can estimate how much effort would be needed to identify such intersecting genes. If the search for intersecting genes returned by the DGEN model during mid development and genes found by previous studies should be possible, the authors could integrate a motivation section to find these intersecting genes within the Introduction section.”*
**Summary** *: “Correlate the output of the DGEN simulation with existing findings of studies addressing similar questions of the causality of the developmental hourglass phenomenon and discuss potential simplifications of the model assumptions that do not fit experimental results or that are not derivable via experiments yet.”*
**Response**: This is a very interesting idea and we thank the reviewers for this suggestion. We are planning to pursue this investigation in a follow-up paper that will also include the previously mentioned analysis of sea-urchin GRNs as well as the DSI/TSI results. We believe that it would be distracting to try to include all these results in this first paper, given that the main focus of this work has been on the computational model and the computational results. We are also open to collaborate in this investigation with the reviewers or other researchers working in this area, if they are interested.
- **Review** *: “The model description is quite intuitive. However, as the validity of the whole study depends on the model parameters chosen, several points need clarification including the rationale and motivation to choose exactly these parameters. One of the most crucial parameters of the model is the regulatory specificity s(l), with 0 <= s(l) <= 1. We think, that adding a more detailed paragraph discussing how s(l) is being estimated, would be highly beneficial due to the importance for the parameter s(l) for the entire model and predictions. In Model-1-4 the authors choose three different values for s(l): In Model-1: s(l) = 0.5 and in Model-2-4: s(l) = l/L, and in Figure 9 a non-linear function s(l) = 0.9 – (0.8 / 1 + exp(\gamma - l) ). A gene g at stage l is modeled to act as upstream regulator for a gene g’ at stage l + 1 with probability s’(l) = 1 – s(l). From this follows that each potential upstream regulator in stage l has the same probability s’(l) to regulate genes g’ in stage l + 1. In other words, Model-1 assigns each potential upstream regulator with the same probability s’(l) = 1 – 0.5 = 0.5 to regulate genes g’ in stage l + 1, in Model-2: s’(l) = 1 – l/L = const., and for the non-linear function s(l): s’(l) = 1 – (0.9 – (0.8 / 1 + exp(\gamma - l) )) = const. It would be advantageous, if the authors could include motivations for the three cut-offs, especially how they derived the sigmoid-like mathematical function (Figure 10, legend caption) they later denote as non-linear function s(l). We do see that modeling the stage specificity with a constant value, an increasing value, and a non-linear function is plausible, but a clear motivation or derivation of the exact values would be beneficial for a better understanding of the modeling process.*
*The authors discuss that their major assumption is that the regulatory specificity increases substantially as development progresses, hence the DGEN becomes gradually sparser along the developmental time axis starting with s(1) ~ 0, … , s(L) ~ 1. Here the authors should include references to experimental observations that support their assumption. Since Models 2-4 rely on this assumption, published experimental studies in line with this assumption need to be referenced. Otherwise, the models applied would seem largely academic.*
*Include a clear statement how the exact values of the stage specificity (regulatory specificity) s(l) have been derived.  State more clearly that s(l) is the crucial parameter determining the observed phenomena.”*
**Response**: The regulatory specificity functions we experimented with are arguably the simplest one could think of. Specifically, we start (Model-1) with the simplest hypothesis that the regulatory specificity does not vary across developmental stages (in the paper we show results for s=0.5 but we have generated results for all values 0.1, 0.2, ... 0.9 and the conclusion remains that when the specificity is constant the network does not evolve into an hourglass shape). Then, we consider (Model-2) a linearly increasing specificity function. We do not argue that this is realistic or that there are experimental results that suggest this linearity or even the increasing trend. It is just the simplest and most parsimonious model that leads to the emergence of an hourglass pattern. Finally, in Figures-9 and 10 we consider a more general specificity function that increases in a non-linear manner, providing a simple way to control the stage at which it gives the mid-range value 0.5. This allows us to examine how the shape of the specificity function s(l) affects the location of the hourglass waist. We hope that follow-up work will reveal experimentally the actual shape of the regulatory specificity function s(l) and there is no doubt that it will not be identical to any of these simple mathematical functions.
  Please note that we have revised the paragraphs about “Model-1” and “Model-2” in the Simulation section to clarify these points.
- **Review**: *“For the probabilities P_{DL} and P_{DP} the explicit formula to compute the corresponding probabilities could also be included within the paragraph analogous to P_{RF}. This would enable a clearer reproducibility.“*
**Response**: Actually there is no formula for the gene deletion and duplication probabilities. They are given probability values that do not depend on any other parameters. At each round of the simulation, a gene is duplicated with probability P_{DP}. If not rewired or duplicated, the gene is deleted with probability P_{DL}.
- **Review**: *“Hourglass score H: Here w(l) is defined as the number of transitioning genes in stage l. In Figure 3 and in section 'Hourglass shape' w(l) is referred to as stage width. In case the definition of w(l) is equivalent to the stage width, it would be beneficial for the reader to see the term stage width in the same sentence as the initial definition of w(l). A correct understanding of the stage width is important to understand the measurement of H.”*
**Response**: The reviewer is right. We will use the “stage width” term when we first introduce the notation w(l).
- **Review**: *“Furthermore, the univariate Mann-Kendall statistic should be referenced to allow a non-statistical community to better understand this way of statistical modeling.”*
**Response**: Please see how we addressed a similar comment (comment #5) in Dmitri Krioukov's review.
- **Review**: *“Transcriptome age index (TAI): In this section the authors describe and perform two different methods to assign each gene a corresponding evolutionary age. For D. melanogaster they collected groups of orthologs from OrthoDB and OrthoMCL, whereas for A. thaliana they obtained phylostratum assignments from Quint et al. 10 that was derived by phylostratigraphy[ref-13.] We do not understand the motivation of merging orthologous gene groups obtained from OrthoDB and OrthoMCL, as both approaches apply different parameters and heuristics to determine orthologous genes. Was this done to maximize the number of potential orthologs to be included in the study? In addition, why did the authors use a different approach for the plant gene set? This seems somewhat inconsistent and statement concerning the plausibility to compare results returned by the DGEN model based on age assignments obtained from phylostratigraphy (A. thaliana) with OrthoDB and OrthoMCL merged orthologs (D. melanogaster) would be appreciated. Furthermore, the database specific parameters they used in OrthoDB and OrthoMCL should be mentioned as well as the database version they used, e.g. OrthoDB2, or OrthoDB3, … , or OrthoDB6.”*
**Summary**: *“Explain why merging OrthoDB results with OrthoMCL results might be plausible and why comparing it with results obtained from phylostratigraphy is reliable.”*
**Response**: The phylostratigraphy technique as employed by Domazet-Loso et al. and Quint et al. involves the assignment of an evolutionary age to each gene in a given species, by tracing the most distant ancestral node containing at least one immediate daughter species with a detectable homologue. A phylogenetic tree of divergent species ranging from cellular organisms transitioning into simple and more complex eukaryota served as a reference to divide phyla radiating from consecutive ancestral points into distinct phylostratum layers. To determine the homologues, BLAST sequence similarity searches were done against complete genomes reliably annotated across the different phylostrata. Similarly, we obtained known homologues of each Drosophila gene from OrthoDB5 and OrthoMCL5 (that also use BLAST based searches) in the species depicted in Figure 4, and mapped them to six different “phylostrata” (marked in Figure 4). Akin to the method described in the above references, we assigned each gene to a specific “phylostratum” based on the farthest detectable homologue of that gene, and this is referred to as “age index” in the paper. This made it possible to compare the age index based results we obtain in Drosophila to the phylostratigraphy based assignments in Arabidopsis.
  Homologues for Drosophila genes were sourced from these two databases, to increase coverage across the species depicted in the tree in Figure 4. Since these two databases use slightly different parameters to identify orthologs, we examined whether the usage of specific databases introduce bias by analyzing data from each database separately and observed that age index of each gene remained the same.
- **Review**: *“ Age index for each stage-pair: There seems to be a typo within the TAI formula. As defined by Domazet-Loso and Tautz ^6^ and adapted to the author’s notation the formula of the TAI should begin TAI(l) not TAI(1).”*
**Response**: We have fixed this problem. Thank you.
- **Review**: *“The authors show based on their findings returned by Model-2-4 that the increasing specificity assumption is the key property behind the developmental hourglass effect. We think that this statement is not clear enough. The authors could phrase more clearly what they mean by “key property behind the hourglass effect” inferred from Model-2-4.”*
**Response**: The reviewer is right. We revised that paragraph as follows:
  “In Model-1 and Model-2, genes can be only removed (due to RW events, potentially followed by RF cascades) and so the average DGEN size decreases as evolutionary time progresses, which is unrealistic. Model-3 and Model-4 are more realistic because they can maintain a roughly constant DGEN size in the long-term. However, as will be shown next, all aspects of the developmental hourglass effect can already be seen with Model-2 (but not with Model-1). This highlights that the increasing specificity assumption is sufficient to generate the hourglass effect. Further, the inclusion of additional biological mechanisms in the model, namely gene duplication and gene deletion, even though they make the model more realistic, they are not necessary for the emergence of the hourglass effect."
- **Review**: *“Age of genes: Here the authors motivate that defining the age of a gene as A(g) = i – t0(g) is plausible, because a rewiring event may give a gene a new function, at least in terms of its upstream and downstream regulators. This statement needs validation from the literature and from experimental studies that already tested this hypothesis for specific rewiring events.”*
**Response**: We have added two references that provide some evidence to the previous hypothesis: Guet *et al.* (2002) and Kim *et al.*(2012).
- **Review**: *“Furthermore, the authors show (Figure 8c) that the evolutionary age at stage l follows the same pattern as the lethality probability: gradually increasing until the waist of the hourglass, and then gradually decreasing. This finding should be correlated with the findings by Galis and Metz ^3^ and should be discussed more clearly in the Discussion.“*
**Response**: The reviewer is right. The findings of Galis and Metz are highly relevant and consistent with our computational results regarding the lethality probability. We modified the paper as follows:
In the Results section:
  “These computational results for the lethality probability across development are consistent with the empirical observations of Galis and Metz \cite{galis2001testing} about the increased mortality of rodents due to perturbations in the phylotypic stage (see Discussion section)."
In the Discussion section:
  “The third prediction of the model is in direct agreement with the empirical observations of Galis and Metz regarding the increased mortality caused by perturbations during the phylotypic stage \cite{galis2001testing}. That study has shown, based on the teratological literature for rodent development, that disturbances in the phylotypic stage lead to much higher mortality than in other stages. Further, such disturbances lead to the co-occurrence of several distinct anomalies in the developing embryo and so the increased mortality cannot be due to a single particularly vulnerable process that takes place at that stage.”
- **Review**: *“In the first sentence of the last paragraph before the Discussion, "… and TAI profiles from Arabidopsis" should be referenced to reference 11 and not 12.“*
**Response**: Thank you, we have fixed this problem.
**Review**: *“Discussion: The authors discuss that the observed pattern of conservation (developmental hourglass) may stem from fundamental organization principles and that the exact origin of these principles remains elusive. Here the authors should include existing studies that also aim at predicting a universal organization principle during development (modularization, etc.) and what exact problems are preventing studies to elucidate this universal phenomenon. We do note, that the authors cite studies that aim to explain the effects causing the early phase of the developmental hourglass ^14 15 16^ and studies aiming to explain effects causing the late phase of the developmental hourglass ^1^, but we think that it should be explicitly stated that the model is designed to test aspects of Raff’s initial hypothesis and that also more recent studies have been investigating the underlying reasons for the pattern in the early phase of the hourglass ^17^.”*
**Response**: The reviewer is right. We have revised the Discussion section to also include a paragraph about Raff’s hypothesis (see our response to comment 1). We have also added another paragraph (see below) to discuss a very recent paper that proposes a different mechanism for the emergence of the hourglass effect.
  “Recently, Friedlander *et al.* have proposed a different mechanism for the emergence of hourglass-like patterns in evolving biological or technological networks, based on a linear system formulation **\cite{friedlander2014evolution}**. They showed that if a system can be represented as a hierarchical and layered linear transformation of an input vector to an output vector, and the desired transformation matrix is rank-deficient, then an evolutionary process that selects that particular transformation can, under certain conditions, converge to an hourglass-like structure. It is not clear yet how to adapt this linear model in the context of inherently non-linear systems, such as gene regulatory networks.”
